# Functional Status of Neuronal Calcium Sensor-1 Is Modulated by Zinc Binding

**DOI:** 10.3389/fnmol.2018.00459

**Published:** 2018-12-14

**Authors:** Philipp O. Tsvetkov, Andrei Yu. Roman, Viktoriia E. Baksheeva, Aliya A. Nazipova, Marina P. Shevelyova, Vasiliy I. Vladimirov, Michelle F. Buyanova, Dmitry V. Zinchenko, Andrey A. Zamyatnin, François Devred, Andrey V. Golovin, Sergei E. Permyakov, Evgeni Yu. Zernii

**Affiliations:** ^1^Aix-Marseille University, CNRS, INP, Institute of Neurophysiopathology, Faculty of Pharmacy, Marseille, France; ^2^Institute of Physiologically Active Compounds (RAS), Chernogolovka, Russia; ^3^Belozersky Institute of Physico-Chemical Biology, Lomonosov Moscow State University, Moscow, Russia; ^4^Institute for Biological Instrumentation of the Russian Academy of Sciences, Pushchino, Russia; ^5^Shemyakin and Ovchinnikov Institute of Bioorganic Chemistry, Russian Academy of Sciences, Pushchino, Russia; ^6^Faculty of Bioengineering and Bioinformatics, Lomonosov Moscow State University, Moscow, Russia; ^7^Institute of Molecular Medicine, Sechenov First Moscow State Medical University, Moscow, Russia; ^8^Faculty of Computer Science, Higher School of Economics, Moscow, Russia

**Keywords:** neuronal calcium sensor-1, zinc, calcium, magnesium, EF-hand motif, dopamine receptor D2R, GRK1, protein aggregation

## Abstract

Neuronal calcium sensor-1 (NCS-1) protein is abundantly expressed in the central nervous system and retinal neurons, where it regulates many vital processes such as synaptic transmission. It coordinates three calcium ions by EF-hands 2-4, thereby transducing Ca^2+^ signals to a wide range of protein targets, including G protein-coupled receptors and their kinases. Here, we demonstrate that NCS-1 also has Zn^2+^-binding sites, which affect its structural and functional properties upon filling. Fluorescence and circular dichroism experiments reveal the impact of Zn^2+^ binding on NCS-1 secondary and tertiary structure. According to atomic absorption spectroscopy and isothermal titration calorimetry studies, apo-NCS-1 has two high-affinity (4 × 10^6^ M^-1^) and one low-affinity (2 × 10^5^ M^-1^) Zn^2+^-binding sites, whereas Mg^2+^-loaded and Ca^2+^-loaded forms (which dominate under physiological conditions) bind two zinc ions with submicromolar affinity. Metal competition analysis and circular dichroism studies suggest that Zn^2+^-binding sites of apo- and Mg^2+^-loaded NCS-1 overlap with functional EF-hands of the protein. Consistently, high Zn^2+^ concentrations displace Mg^2+^ from the EF-hands and decrease the stoichiometry of Ca^2+^ binding. Meanwhile, one of the EF-hands of Zn^2+^-saturated NCS-1 exhibits a 14-fold higher calcium affinity, which increases the overall calcium sensitivity of the protein. Based on QM/MM molecular dynamics simulations, Zn^2+^ binding to Ca^2+^-loaded NCS-1 could occur at EF-hands 2 and 4. The high-affinity zinc binding increases the thermal stability of Ca^2+^-free NCS-1 and favours the interaction of its Ca^2+^-loaded form with target proteins, such as dopamine receptor D2R and GRK1. In contrast, low-affinity zinc binding promotes NCS-1 aggregation accompanied by the formation of twisted rope-like structures. Altogether, our findings suggest a complex interplay between magnesium, calcium and zinc binding to NCS-1, leading to the appearance of multiple conformations of the protein, in turn modulating its functional status.

## Introduction

Divalent metal ions play a vital role in the vast majority of cellular processes. Among them, two alkaline earth metals, magnesium and calcium, as well as the transition metal zinc, are particularly important, since they are the most abundant ones in the human body. These ions are significantly different in intracellular levels and play different physiological roles. While intracellular concentration of free magnesium is high (0.5–2 mM), the free concentration of calcium and zinc is much lower (40–100 nM and 100 pM, respectively) (Romani and Scarpa, 1992;Sabatini et al., 2002;Krezel and Maret, 2006). In addition, free calcium concentration drastically increases in signaling waves, reaching > 100 μM in magnitude (Augustine et al., 2003). Consistently, magnesium binds to proteins with much lower affinity (equilibrium association constant, K_a_, below 10^5^ M^-1^) than calcium (*K*_a_> 10^5^ M^-1^) and zinc (*K*_a_ > 10^7^ M^-1^) (for review, see (Dudev and Lim, 2003)). Magnesium binding to proteins is less specific compared to Ca^2+^ and Zn^2+^ binding; and, although the common structural motif for Mg^2+^ binding has not been described yet, magnesium is able to occupy calcium and zinc-binding sites. Yet, at high concentrations, zinc can compete with other metals or bind to nonspecific sites in proteins, which may interfere with their structural integrity and normal function, thus contributing to pathology (Choi and Koh, 1998;Barwinska-Sendra and Waldron, 2017).

The great majority of calcium-binding proteins (CaBPs) contain the same helix-loop-helix Ca^2+^-binding motif (referred to as an “EF-hand”), wherein calcium ion is coordinated by six oxygen atoms. Generally, CaBPs comprise several EF-hands, which bind calcium with micromolar dissociation constant. Commonly, this interaction induces a significant rearrangement in the CaBP structure, inducing the exposure of its hydrophobic surface, which is responsible for interaction with target(s), resulting in their activation/deactivation. All CaBPs have specific tissue, cellular and subcellular distribution profiles (for review, see (Yanez et al., 2012)). Along with other tissues, CaBPs are abundantly expressed in the nervous system where they play an essential role in decoding calcium signals and regulating many processes crucial for the viability and functioning of neurons. The calcium signals, emanating from photoreceptor neurons in response to light stimuli, regulate rhodopsin desensitization and cGMP synthesis through interaction with recoverin and guanylate cyclase activating proteins (GCAPs), two members of the neuronal calcium sensor (NCS) family [reviewed in (Burgoyne, 2007;Burgoyne and Haynes, 2012;Koch and Dell’Orco, 2015)]. Neuronal calcium sensor-1 (NCS-1) is another NCS protein, which was found in photoreceptors and other retinal neurons (De Raad et al., 1995;Baksheeva et al., 2015). In contrast to recoverin and GCAPs, NCS-1 is expressed throughout the nervous system. Its N-terminus contains a myristoyl group, which participates in the interaction of the protein with cellular membranes. According to *in vitro* and *in vivo* data, NCS-1 regulates more than 20 target proteins, including G protein-coupled receptors and their kinases (GRKs). As such, NCS-1 participates in neuronal growth and survival, reception, neurotransmission, synaptic plasticity and other cellular mechanisms [for review, see (Burgoyne and Haynes, 2012)]. NCS-1 contains four EF-hand motifs, but only three of them (EF2, EF3 and EF4) are able to bind calcium with nanomolar to micromolar dissociation constants (Jeromin et al., 2004). In the absence of calcium, two EF-hands (EF2 and EF3) could be occupied by magnesium ions (Warren et al., 2007;Aravind et al., 2008).

Zinc is long known to be a key element in neuronal growth and activity necessary for the normal development and functioning of the brain (Frederickson et al., 2005). Zinc deficiency results, for instance, in lowered glutamate receptor expression and decreased cognitive and motor performance in children (Penland et al., 1997;Gardner et al., 2005). It is also critical for the development, viability and specific function of the retinal neurons (Ugarte and Osborne, 2014). The levels of Zn^2+^ in the retina and retinal pigment epithelium are decreased in the elderly, which may contribute to the pathogenesis of age-related macular degeneration (Wills et al., 2008;Lyubartseva and Lovell, 2012). Expression of retinal proteins involved in zinc homeostasis also becomes downregulated with age (Leung et al., 2012). In general, zinc serves to maintain the structure and function of hundreds of proteins, including enzymes of all known classes, transcription factors, receptors and signaling proteins. While, in many cases, zinc is tightly bound to proteins, it could also interact with their Zn^2+^-binding sites transiently in order to conduct biochemical stimuli. The most abundant Zn^2+^-binding motif in proteins is the Z-finger, which chelates zinc ion with nanomolar to picomolar affinity [for review, see (Maret and Li, 2009)]. Nevertheless, zinc ions can bind to EF-hands [as reported for calmodulin (Warren et al., 2007)] or between two EF-hand motifs, or even in-between two protein subunits in dimer, as observed in some members of the S100 family (Tsvetkov et al., 2010;Moroz et al., 2011). Considering that some members of the NCS family, such as recoverin (Permyakov et al., 2003), are able to bind zinc ions, we hypothesized that NCS-1 is also sensitive to Zn^2+^.

In this study, we demonstrate that apo-NCS-1 has two high-affinity zinc-specific sites and one low-affinity zinc-binding site. Zinc binding to NCS-1 reduces stoichiometry and increases the affinity of Ca^2+^ binding to the protein. In contrast, physiologically relevant Mg^2+^- and Ca^2+^-loaded NCS-1 forms only bind two zinc ions with high affinity, which stabilizes their structure and may be required for maintaining the functional status of these forms. In addition, our findings suggest that the elevated concentration of free zinc, characteristic of some neurodegenerative and neuro-ophthalmological disorders, may lead to the formation of unstable, prone-to-aggregation pathological NCS-1 forms.

## Materials and Methods

### Purification of Proteins and Membranes

NCS-1 was obtained according to the protocol previously developed for recoverin with some modifications. To obtain recombinant myristoylated protein, NCS-1 gene was co-expressed in *Escherichia coli* strain BL21(DE3) Codon Plus RP with N-myristoyl transferase 1 from *Saccharomyces cerevisiae* (4 h, 37°C) in the presence of 200 μg/ml myristic acid. The cells were harvested by centrifugation and lysed by freezing/thawing in extraction buffer (50 mM Tris-HCl (pH 8.0), 100 mM NaCl, 1 mM EDTA, 1 mM PMSF, 1 mM DTT) and subsequent incubation in the presence of 50 μg/ml of egg white lysozyme in the same buffer for 30 min. The lysate was clarified by centrifugation, loaded onto Phenyl Sepharose column (GE Lifesciences) equilibrated with 20 mM Tris-HCl buffer (pH 8.0), 2 mM CaCl_2_, 1 mM DTT and NCS-1 was eluted using the same buffer containing 2 mM EGTA. The obtained protein was loaded on HiTrap Q FF anion exchange column (GE Lifesciences) equilibrated with 20 mM Tris-HCl buffer (pH 8.0), 1 mM DTT and eluted by linear gradient of 0-1 M NaCl in the same buffer. NCS-1 (> 90% purity) was present in the fractions eluted at 380-500 mM NaCl. The obtained protein was dialysed overnight against 20 mM Tris (pH 8.0), 1 mM DTT and stored at -70°C. Alternatively, to remove residual calcium NCS-1 sample obtained immediately after anion exchange chromatography was subjected to dialysis against 50 mM Tris-HCl (pH 8.0), 5 mM EDTA (3 h), followed by dialysis against deionized water (3 h), and dialysis against 20 mM Tris-HCl (pH 8.0), 1 mM DTT (Blachford et al., 2009). The degree of NCS-1 myristoylation was determined by analytical HPLC using a reversed-phase column [Phenomenex Luna C18(2)] and was more than 97%. NCS-1 concentration was measured with bicinchoninic acid assay kit (Thermo Fisher Scientific) or spectrophotometrically using previously determined molar extinction coefficient at 280 nm of 21,430 M^-1^ (Kazakov et al., 2017).

N-terminal fragment of GRK1 (M1-G183) was obtained as GST-fusion construct (GST-N-GRK1) following the previously developed procedure (Komolov et al., 2009).

Dopamine receptor D2 (D2R) peptide (N430-R443) was produced using Fmoc/But solid-phase peptide synthesis.

Polyclonal (monospecific) antibodies against NCS-1 were generated by rabbit immunization and purified from immune serum on a column with immobilized antigen according to the previously published procedure (Zernii et al., 2003).

Photoreceptor membranes were prepared from frozen bovine retinas following the standard protocol with some modifications described in (Grigoriev et al., 2012).

### Equilibrium Dialysis Experiments

Ca^2+^/Zn^2+^ binding to NCS-1 was studied by equilibrium dialysis method using 96-well micro-equilibrium dialysis system (HTDialysis, LLC) (Banker et al., 2003; Waters et al., 2008). Each well (500 μL) of the teflon block was separated by dialysis membrane (regenerated cellulose, 3.5 kDa MWCO). One half of each well was filled with 180 μL of (4.4–6.2) μM solution of NCS-1 in a buffer (10 mM Hepes-KOH, pH 7.6), whereas the other half contained 180 μL of the same buffer with (2–150) μM Zn(NO_3_)_2_ or (2–50) μM Ca(NO_3_)_2_ without NCS-1. The wells were tightly sealed and equilibrated by continuous shaking (130 rpm) of the block at (25.0 ± 0.5)°C for 17–20 h. Total concentrations of Ca^2+^/Zn^2+^ in the equilibrated solutions were measured by electrothermal atomization atomic absorption spectrometer iCE 3000 (Thermo Scientific), using argon as an inert gas. Zinc content was evaluated using the absorption bands at 213.9 or 307.6 nm and deuterium background correction. For calcium content estimates, the band at 422.7 nm and Zeeman background correction were used. The analytical signal was calibrated using AAS standard solutions for Ca^2+^ (Sigma-Aldrich #69349) and Zn^2+^ (Sigma-Aldrich #18827). Concentration of Ca^2+^/Zn^2+^ bound to NCS-1 was estimated for each well as a difference between the total metal concentrations measured for both halves of the well, assuming that free Ca^2+^/Zn^2+^ concentrations do not differ between the two halves of the well.

### Analytical Gel-Filtration

Analytical gel-filtration of NCS-1 forms was carried out using fast protein liquid chromatography instrument as described for GCAP2 in (Olshevskaya et al., 1999) with modifications. NCS-1 (180 μM) was pre-incubated for 30 min at 37°C in 50 mM Tris-HCl buffer (pH 8.0), 150 mM NaCl, 1 mM DTT containing either 1 mM EGTA or 1 mM Ca^2+^ or 100 μM Zn^2+^. The obtained protein sample (200 μl) was loaded onto Superdex 200 10/300 GL column (GE Lifesciences) pre-equilibrated with the same buffer and eluted at 0.5 ml/min. Alternatively, gel-filtration was performed using high performance liquid chromatography instrument on Ultropack TSK G 2000 SW column (Pharmacia) at 1 ml/min.

### Isothermal Titration Calorimetry (ITC)

Binding of divalent ions (Zn^2+^, Ca^2+^, and Mg^2+^) to NCS-1 was analyzed by ITC using MicroCal iTC200 instrument as described previously (Tsvetkov et al., 2012, 2013). Experiments were performed at 25°C in 50 mM Tris-HCl buffer (pH 7.5) in the presence of 1 mM TCEP. Protein concentration in the calorimetric cell was 25 μM, whereas the concentration of ions in the syringe varied from 375 to 750 μM. In competition experiments the concentration of competitive ions (Zn^2+^, Ca^2+^, and Mg^2+^) in cell and syringe were 250 μM, 1 mM and 5 mM, respectively. NCS-1 was titrated by repeated injections of 2 μL aliquots of ions solution. If necessarily syringe was refilled with the same solution without cell refilling and the titration was continued. Each resulting titration peak was integrated and plotted as a function of the NCS-1/ion molar ratio. The baseline was measured by injecting titrant into the protein-free buffer solution. Data were analyzed using Origin software and were fitted with “sequential binding,” “one set of sites” or “two set of sites” models via a non-linear least squares minimization method and led to the determination of affinity constants (*K*_a_), enthalpy changes (ΔH) and stoichiometry. Thermodynamic values are an average of at least three different experiments.

Binding of NCS-1 to D2R peptide was registered using MicroCal VP-ITC instrument, according to previously developed protocol (Pandalaneni et al., 2015) with modifications described in (Vladimirov et al., 2018). Experiments were performed at 25°C in 20 mM Tris-HCl buffer (pH 8.0), 150 mM NaCl, 1 mM EDTA. Alternatively, the buffer contained 5 mM CaCl_2_ instead of EDTA, with or without addition of 100 μM ZnCl_2_. Recombinant NCS-1 was dialyzed against the same buffer, and protein concentration of the stock solution was adjusted to 1 mM. Calorimetric cell contained 50 μM peptide, which was titrated by thirty 10 μl injections of NCS-1. Each injection was followed by 5 min stabilization phase. The resulting titration peaks were integrated and plotted as a function of the NCS-1/ion molar ratio. The baseline was measured by injecting the protein into the working buffer solution. Data were analyzed using Origin software and were fitted with “one set of sites” model. Thermodynamic parameters were determined as an average of at least three different experiments.

### Fluorimetry and Light Scattering (LS)

Fluorescence emission spectra of NCS-1 and bis-ANS were measured using Cary Eclipse spectrofluorimeter (Varian Inc.), equipped with a Peltier-controlled cell holder essentially as previously described (Baksheeva et al., 2015; Zernii et al., 2015). Tryptophan fluorescence of NCS-1 (14 μM) was excited at 280 nm and measured at 25°C in 10 mM Hepes-KOH, 100 mM KCl, pH 7.6 buffer under various content of metal ions: either metal-free conditions (1 mM EDTA) or in the presence of Mg^2+^ (1 mM MgCl_2_), Ca^2+^ (100 μM CaCl_2_) or Zn^2+^ (100 μM ZnCl_2_), or their combinations. Fluorescence of bis-ANS (1 μM) complexes with NCS-1 (5 μM) in the same buffer at 20^o^C was excited at 385 nm. All spectra were corrected for spectral sensitivity of the instrument and fitted to log-normal curves (Burstein and Emelyanenko, 1996) using LogNormal software (IBI RAS, Pushchino, Russia). Spectrofluorimetric temperature scans were performed at the average heating rate of 0.5°C/min. The mid-transition temperatures for conversion from the native to the thermally denatured protein state were estimated from fits of the temperature dependencies of λ_max_ by Boltzmann function using OriginPro 9.0 software (OriginLab Corporation, United States).

Alternatively, tryptophan fluorescence and thermal stability of NCS-1 in the presence of different concentrations of divalent ions were measured in 50 mM Tris-HCl, 1 mM TCEP, pH 7.5 buffer using differential scanning fluorimetry (DSF) instrument Prometheus NT.Plex (NanoTemper Technologies) equipped with LS module. NanoDSF grade capillaries were filled with 25 μM NCS-1 solution. Concentrations of Zn^2+^, Ca^2+^ or Mg^2+^ varied from 25 to 500 μM. The capillaries were loaded into the Prometheus NT.Plex instrument and the ratio of NCS-1 fluorescence emission intensities at 330 nm (*I*_330_) and 350 nm (*I*_350_) was registered at 25°C at low detector sensitivity and excitation power of 10% (excitation wavelength of 280 nm). Then capillaries were heated from 15°C to 95-110°C at rate of 1 K/min. The unfolding mid-transition temperature (*T*_m_) was determined from first derivative of the temperature dependence of the ratio, as implemented in Prometheus NT.Plex software. The temperatures of protein aggregation (*T*_agg_) were determined from temperature dependences of the LS at 350 nm.

### Circular Dichroism (CD)

Circular dichroism measurements were carried out with a JASCO J-810 spectropolarimeter (JASCO Inc., Japan), equipped with a Peltier-controlled cell holder as described in ref. (Permyakov et al., 2012). Briefly, CD spectra of NCS-1 (8 μM) were recorded at 25^o^C in 10 mM Hepes-KOH, 100 mM KCl, pH 7.6 buffer, either under metal-free conditions (1 mM EDTA) or in the presence of Mg^2+^ (1 mM MgCl_2_), Ca^2+^ (100 μM CaCl_2_) or Zn^2+^ (100 μM ZnCl_2_), or their combinations. The secondary structure contents were estimated using CDPro software package (Sreerama and Woody, 2000).

### Modeling and QM/MM Molecular Dynamics

To predict zinc binding sites in NCS-1, the major parameters for Zn^2+^ coordination (distance and angle between the cation, coordinator and one of following atoms) were analyzed in 6327 structures available in PDB. The possible range of coordinators was defined as a list of the following types of atoms: SG, ND1, NE2, OD1, OD2, OE1, OE2, OG, OG1, OH and backbone oxygen (O). The maximal distance from the cation to chelator was limited at 3Å (Laitaoja et al., 2013). Based on these data, a search for possible Zn^2+^-binding sites was performed in X-ray structure of NCS-1 (PDB 5AEQ, Pandalaneni et al., 2015) starting from identification of tightly interconnected (can be defined as cliques in undirected graph) zinc coordinators, namely at least 3 atoms at distances less than 6Å. For every found coordinator, all possible positions of zinc were predicted yielding local density areas. The positions that fall within VdW radii of neighboring atoms were subtracted. The resulting putative Zn^2+^-binding areas were ranged according to maximum density, which was visualized as volumetric data in PyMol (DeLano, 2002).

Putative coordination of zinc in the identified areas of Ca^2+^-occupied EF-hands was assessed by evaluating cation coordination stability using QM/MM molecular dynamics simulations. Two best score metal binding sites at distance of more than 3Å from each other were selected in Ca^2+^-binding loops of all three EF-hands. The best site was loaded with Ca^2+^, whereas the second site was loaded with Zn^2+^. The resulting three systems were filled with TIP3P water with 0.1 M NaCl and the net charge was neutralized with additional ions. The solvated protein, Ca^2+^ and Zn^2+^ were positionally restrained and water together with sodium and chloride ions were equilibrated with molecular dynamics simulation for 100 ps. On the next step, each simulation system was divided in molecular mechanics (MM) and quantum mechanics (QM) subsystems. MM subsystem was described with parameters from the parm99sb-ildn force field with corrections (Lindorff-Larsen et al., 2010). The QM subsystem was described utilizing DFTB approach (Grimme et al., 2010; Gaus et al., 2012) and defined as any atom including waters at distance less than 5Å from Zn^2+^ or Ca^2+.^ The coupling between MM and QM subsystems was performed using ONIOM approach (Dapprich et al., 1999). Linking atoms were introduced in single C-C bonds to preserve unsaturated structures in QM systems. The accordingly prepared systems were subjected to the QM/MM molecular dynamics simulation in NVT ensemble. The time step was set to 0.2 fs. Temperature coupling was performed with Velocity Rescale scheme (Bussi et al., 2007) allowing observing behavior of the systems at 300K. The total length of each simulation was set to 30 ps. All simulations were performed with GROMACS/DFTB package (Abraham et al., 2015; Kubař et al., 2015).

### Equilibrium Centrifugation Assay

The binding of NCS-1 to urea-washed photoreceptor membranes was performed according to the previously described procedure (Weiergräber et al., 2006; Senin et al., 2011) with some modifications. Briefly, 25 μM NCS-1 in buffer containing 20 mM Tris (pH 8.0), 150 mM NaCl and saturating concentration of MgCl_2_ (20 mM), was mixed with the membranes in the absence and in the presence of 1 mM CaCl_2_ and 0–100 μM ZnCl_2_, agitated in a thermostatic shaker for 15 min (37°C, 1000 rpm) and centrifuged (24000 × *g*, 15 min). The pellet was dissolved in SDS-PAGE buffer and the rate of NCS-1 binding to membranes was measured by densitometric analysis of bands in polyacrylamide gel, using GelAnalyzer software^1^.

### Pull-Down Assay

Interaction of Zn^2+^-bound NCS-1 with GST-tagged N-terminal fragment of GRK1 (M1-G183) was monitored using analytical affinity chromatography (pull-down assay) (Zernii et al., 2011). Briefly, 50 μg of the fusion protein was immobilized on Glutathione Sepharose resin in 20 mM Tris (pH 8.0), 150 mM NaCl, 1 mM DTT. Next, 25 μM of NCS-1 was applied to the pellet. This suspension was incubated in a thermostatic shaker (1000 rpm) for 1 h at 4°C in the presence of 1 mM CaCl_2_ and 0–100 μM ZnCl_2_. After each incubation step non-bound protein was removed by washing the resin with the working buffer containing 0.05% Tween 20. Bound NCS-1 was eluted by SDS-PAGE sample buffer and analyzed by western blotting.

### Precipitation Assay

Precipitation of NCS-1 was monitored in the mixture containing 25 μM protein, 20 mM Tris (pH 8.0), 150 mM NaCl, 1 mM DTT, and 0–500 μM ZnCl_2_ with or without addition of 1 mM CaCl_2_. NCS-1 was incubated for 30 min at 37°C with mild agitation, then precipitated protein was collected by centrifugation (24000 × *g*, 15 min) and the pellet was dissolved in SDS-PAGE sample buffer and analyzed by SDS-PAGE. The ratio of precipitated NCS-1 was estimated by densitometric analysis.

### Transmission Electron Microscopy (TEM)

Four microliters of NCS-1 samples (10 μM) obtained in the presence of 1 mM ZnCl_2_ were placed on carbon-coated copper grids (300 mesh) during 1 min. After having been blotted, grids were washed with distilled water, blotted again, negatively stained for 30 s with 2% (wt/vol) uranyl acetate. The grids were then dried and observed with a JEOL 2200FS transmission electron microscope (Tokyo, Japan) operating at 200 kV. Images were recorded using a 4k × 4k slow-scan CCD camera (Gatan, Inc., Pleasanton, United States).

## Results

### Stoichiometry of Zinc Binding to NCS-1

**FIGURE 1:**
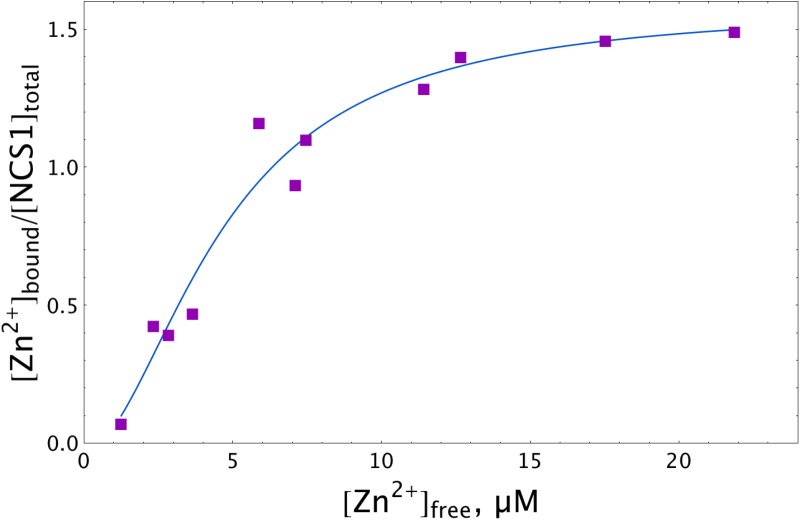
Zn^2+^ binding to NCS-1 according to equilibrium dialysis experiments. The NCS-1 (5 μM) sample was pre-equilibrated with (2–150) μM Zn^2+^ by equilibrium dialysis (3.5 kDa MWCO) at 25°C. The concentrations of NCS-1-bound zinc ([Zn^2+^]_bound_) and free zinc ([Zn^2+^]_free_) in the resulting solutions were measured by electrothermal atomization AAS, using the absorption bands at 213.9 nm or 307.6 nm. The solid curve approximates the experimental data by Hill equation.

To test the hypothesis of the zinc interaction with NCS-1, we directly assessed the amount of zinc ions that can be bound per protein molecule using a micro-equilibrium dialysis system. To this end, a sample of recombinant myristoylated NCS-1 was prepared by Ca^2+^-dependent hydrophobic and ion exchange chromatographies and subjected, at 25°C, to 20-h dialysis (MWCO of 3.5 kDa) against buffer containing different Zn^2+^ concentrations. Zinc concentrations on both sides of the dialysis membrane were then measured by electrothermal atomization atomic absorption spectroscopy (AAS). The approximation of the resulting experimental data using the Hill equation (Figure 1) revealed half-maximal binding at 4.7 μM [Zn^2+^]_free_. Meanwhile, maximal stoichiometry of the zinc binding reached 1.5. The fractional stoichiometry may have been due to either the manifestation of an intermolecular zinc-binding site (which suggests NCS-1 multimerization) or the inaccessibility to zinc ions for some fraction of the protein molecules. Analytical gel filtration experiments did not reveal NCS-1 multimers in the presence of Zn^2+^ (data not shown). Moreover, under these solution conditions, NCS-1 exhibited an even lower Stokes radius than in the presence of Ca^2+^ or EGTA (which also confirms zinc-binding to NCS-1). Therefore, we supposed that some fraction of the zinc-binding sites of NCS-1 remain shielded from zinc. Since AAS analysis of the NCS-1 sample revealed a calcium-to-protein molar ratio of 0.42, we suggest that at least one of zinc-binding sites of NCS-1 overlapped with its active EF-hands. Therefore, we further decalcified NCS-1 samples by stepwise dialysis against EDTA, deionized water and a reaction buffer, as described earlier for the non-myristoylated protein (Blachford et al., 2009). As evidenced by AAS, this procedure decreased the fraction of residual calcium in NCS-1 sample down to 0.17, which means that the protein remains Ca^2+^-bound only by 5.6% of the saturation. Although the resulting protein sample (apo-NCS-1) bound Ca^2+^ with a stoichiometry of 3 (data not shown), we failed to detect zinc binding to apo-NCS-1 by AAS, as its long-term incubation during equilibrium dialysis in the presence of Zn^2+^ was accompanied by NCS-1 aggregation and accumulation on the dialysis membrane. Given this observation, further characterization of the cation-binding properties of NCS-1 was performed by ITC. Yet, the AAS data represent direct evidence of zinc binding to NCS-1, revealing its dependence on calcium binding.

### Thermodynamics of Calcium and Magnesium Binding to NCS-1

**FIGURE 2:**
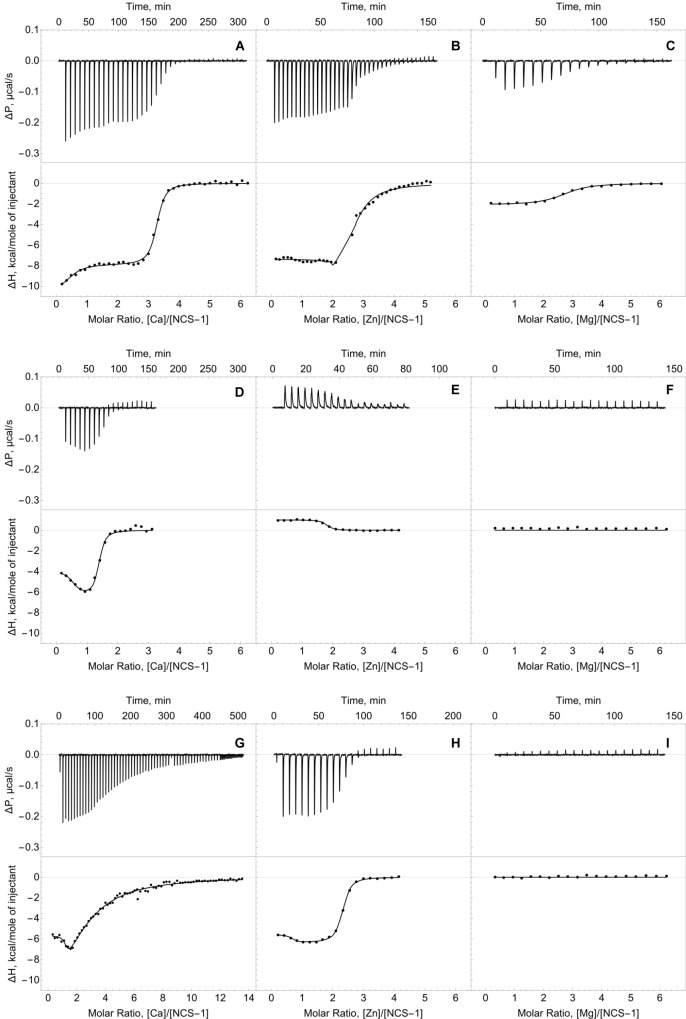
Thermodynamics of calcium, zinc and magnesium binding to NCS-1. Typical ITC curves (upper panels) and binding isotherms (lower panels) representing titration of NCS-1 (25 μM) by different cations. **(A)** Binding of Ca^2+^. **(B)** Binding of Zn^2+^. (**C**) Binding of Mg^2+^. **(D)** Binding of Ca^2+^ in the presence of 250 μM Zn^2+^. **(E)** Binding of Zn^2+^ in the presence of 1 mM Ca^2+^. **(F)** Binding of Mg^2+^ in the presence of 250 μM Zn^2+^. **(G)** Binding of Ca^2+^ in the presence of 5 mM Mg^2+^. **(H)** Binding of Zn^2+^ in the presence of 5 mM Mg^2+^. **(I)** Binding of Mg^2+^ in the presence of 1 mM Ca^2+^. Best fits are shown as solid curves (see Table 1).

We employed ITC to determine Ca^2+^/Mg^2+^-binding parameters of the decalcified myristoylated NCS-1 (apo-NCS-1) sample, given that previous data on these properties were contradictory. Apo-NCS-1 (25 μM) was titrated by CaCl_2_ in 50 mM Tris-HCl pH 7.5 buffer in the presence of 1 mM TCEP (Figure 2A, top panel). The use of “one set of sites” or “two sets of sites” models did not allow for a correct fit of the titration curve. Meanwhile, the experimental data were well fitted using the “sequential binding” model assuming three calcium sites (Figure 2A, bottom panel): the respective equilibrium association constants are 4.3 × 10^6^ M^-1^, 2.0 × 10^5^ M^-1^, and 3.5 × 10^6^ M^-1^ (Table 1). It should be noted that calcium binding to the two high-affinity sites was enthalpy-driven (Table 1), while calcium binding to the lower affinity site had an unfavorable enthalpy of 1.4 kcal/mol, indicating significant rearrangement of hydrophobic amino acids upon calcium binding to this site.

**Table 1:** Thermodynamic parameters of zinc, calcium and magnesium binding to NCS-1 in 50 mM Tris-HCl buffer (pH 7.5) in the presence of 1 mM TCEP at 25°C, estimated from ITC data (see Figure 2).

Ion	Competitor	K_A_^1^, M^-1^	ΔH^1^, kcal M^-1^	K_A_^2,^ M^-1^	ΔH^2^, kcal M^-1^	K_A_^3^, M^-1^	ΔH^3^, kcal M^-1^
Ca^2+∗^	–	4.3 × 10^6^	-10.1	2.0 × 10^5^	1.4	3.5 × 10^6^	-17.8

**Ion**	**Competitor**	**N^1^**	**K_a_^1^, M^-1^**	**ΔH^1^, kcal M^-1^**	**N^2^**	**K_a_^2^, M^-1^**	**ΔH^2^, kcal M^-1^**

Ca^2+∗∗^	Zn^2+^	0.6	5.9 × 10^7^	-3.9	0.9	3.5 × 10^6^	-6.5
	Mg^2+^	1.5	2.2 × 10^4^	-17.6	1.2	4.3 × 10^6^	-5.7
Zn^2+∗∗^	–	0.7	2.3 × 10^5^	-11.8	2.0	9.2 × 10^6^	-7.3
	Ca^2+^				1.7	2.9 × 10^6^	1.0
	Mg^2+^				1.7	4.2 × 10^6^	-6.3
Mg^2+∗∗^	–				2.7	5.2 × 10^5^	-2.1
	Ca^2+^	No binding					
	Zn^2+^	No binding					


*^∗^Data were fitted using “sequential binding” model. ^∗∗^Data were fitted using “two sets of sites” model. The concentrations of competitive ions Zn^*2*+^, Ca^*2*+^, and Mg^*2*+^ were 250 μM, 1 mM, and 5 mM, respectively.*

The ITC data on magnesium binding to apo-NCS-1 were well described by the “one set of sites” model (Figure 2C), revealing 2.7 Mg^2+^ bound per protein molecule with an equilibrium association constant of 5.2 × 10^5^ M^-1^ (Table 1). Considering that apo-NCS-1 contained small fraction of residual calcium (0.17, see previous section), one can suppose that actual stoichiometry of Mg^2+^ binding tends to 3. Indeed, magnesium ions compete with calcium for the same sites as no Mg^2+^ binding was observed for NCS-1 saturated with Ca^2+^ (1 mM). Consistent with this suggestion, in the presence of 5 mM Mg^2+^ NCS-1 exhibited decreased affinity with calcium (Table 1). At the same time, the number of Ca^2+^ bound per NCS-1 molecule in excess of magnesium reached 2.7, indicating that calcium completely replaced the bound magnesium ions.

### Thermodynamics of Zinc Binding to NCS-1

The apo-form of myristoylated NCS-1 (25 μM) in 50 mM Tris-HCl pH 7.5 buffer, in the presence of 1 mM TCEP, was titrated by ZnCl_2_ using ITC (Figure 2B). The fitting of the resulting titration curve using the “two sets of sites” model indicated that NCS1 bound Zn^2+^ in two equal high-affinity sites and one low-affinity site. The corresponding equilibrium association constants were calculated as 9.2 × 10^6^ M^-1^ and 2.3 × 10^5^ M^-1^, respectively (Table 1). The saturation of NCS-1 with Ca^2+^ or Mg^2+^ abolished the low-affinity Zn^2+^ binding, while zinc affinity of the other two sites was almost unaffected. Meanwhile, the enthalpy changes (ΔH) accompanying zinc interaction with these forms differed: ΔH was negative for Zn^2+^ binding to apo- and Mg^2+^-loaded NCS-1, and positive for Zn^2+^ binding to Ca^2+^-loaded NCS-1, thereby reflecting significant conformational differences between Mg^2+^- and Ca^2+^-loaded NCS-1 states. Finally, Zn^2+^-loaded NCS-1 was unable to bind magnesium, but coordinated 1.5 Ca^2+^ ions per protein molecule with increased affinity of the first site (*K*_a_ = 5.9 × 10^7^ M^-1^).

Overall, the different modes of zinc binding to NCS-1, as revealed by our data, indicate that structural and functional consequences of this interaction depend on NCS-1 conformation. Thus, the ITC data argue for the existence of Mg^2+^-bound, Ca^2+^-bound and Zn^2+^-bound conformers of NCS-1 as well as its Zn^2+^(Mg^2+^)-bound, Zn^2+^(Ca^2+^)-bound and Ca^2+^(Zn^2+^)-bound forms, where Zn^2+^ or Ca^2+^ are bound to the background of the excess of Mg^2+^, Ca^2+^ and Zn^2+^, respectively (hereinafter the cation that is taken in excess is indicated in parentheses).

### Conformational Properties of Zinc-Bound NCS-1

The ITC experiments suggested the existence of several distinct states of NCS-1 with two different metal ions bound simultaneously. To explore structural differences between these NCS-1 states, we measured the intrinsic fluorescence spectra of NCS-1 in the presence of various combinations of the metals studied, which enabled examination of the mobility and polarity of the microenvironment of Trp30 and Trp103 residues located in N- and C-terminal domains of the protein.

**FIGURE 3:**
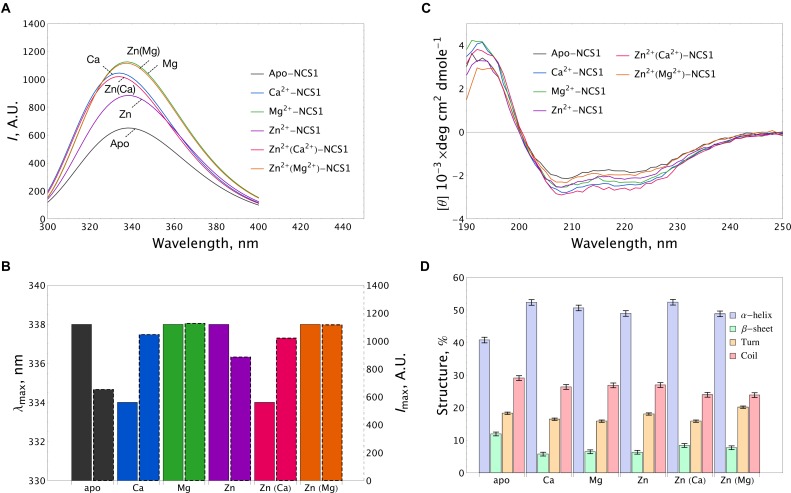
Conformational properties of NCS-1 under saturating concentrations of calcium, magnesium and zinc. **(A)** Representative tryptophan fluorescence spectra of 14 μM NCS-1 under metal-free conditions (1 mM EDTA) or in the presence of Ca^2+^ (100 μM CaCl_2_), Mg^2+^ (1 mM MgCl_2_), Zn^2+^ (100 μM ZnCl_2_) or their combinations. **(B)** The histogram demonstrating maximum position λ_max_ (left bar) and maximal intensity *I*_max_ (right bar) of fluorescence spectrum for the data shown in panel **(A)**. **(C)** Representative CD spectra of 8 μM NCS-1 under metal-free conditions or in the presence of Ca^2+^ (100 μM CaCl_2_), Mg^2+^ (1 mM MgCl_2_), Zn^2+^ (100 μM ZnCl_2_) or their combinations. **(D)** The histogram representing secondary structure contents (in %) for each NCS-1 spectrum shown in panel **(C)**.

The fluorescence spectra of 15 μM NCS-1 were measured either under metal-free conditions (1 mM EDTA) or in the presence of 1 mM Mg^2+^, 0.1 mM Ca^2+^ or 0.1 mM Zn^2+^, or their combinations. Apo-NCS-1 exhibited a characteristic tryptophan fluorescence emission spectrum with a maximum position (λ_max_) at 338 nm (Figures 3A,B). Mg^2+^ binding significantly increased the maximal intensity (*I*_max_) of the fluorescence emission spectrum of NCS-1 without affecting the maximum position. Calcium binding to NCS-1 increased its *I*_max_ value and shifted its λ_max_ to 334 nm, indicating movement of the emitting Trp residue(s) to a less polar and/or mobile environment. Zinc binding to apo-protein increased *I*_max_ without affecting λ_max_ resembling the effect of magnesium in this respect (Figures 3A,B). Meanwhile, the presence of zinc only moderately affected the fluorescence spectra of Mg^2+^- and Ca^2+^-saturated NCS-1, indicating minor structural rearrangements near to the emitting Trp residue(s) under these experimental conditions.

Apo-NCS-1 (8 μM) represented far-UV circular dichroism (CD) spectra typical for an α-helical fold with characteristic minima at 208 nm and 222 nm (Figure 3C). Binding of all examined cations, including zinc, to NCS-1 was accompanied by a similar increase in its α-helical content and a decrease in the content of β-sheets and unordered regions (Figure 3D and Supplementary Table S1). Importantly, NCS-1 contains only two short antiparallel β-sheets, which connect the Ca^2+^-binding loops of EF1-EF2 and EF3-EF4 pairs of EF-hand motifs (Heidarsson et al., 2012; Pandalaneni et al., 2015). Therefore, the revealed similar changes in the β-structure content of NCS-1 upon binding of Ca^2+^/Mg^2+^ and Zn^2+^ suggest that zinc binds to sites overlapping with the EF-hand loops. It should be noted that single and double ion-bound NCS-1 forms also exhibited certain differences in their secondary structure (Figure 3D and Supplementary Table S1). Thus, Ca^2+^-bound and Zn^2+^(Ca^2+^)-bound forms, as well as Mg^2+^-bound and Zn^2+^(Mg^2+^)-bound forms, were identical in α-helical content, but differed in β-structure content 1.46-fold and 1.27-fold, respectively (Supplementary Table S1). These data suggest that zinc binding to Ca^2+^-saturated or Mg^2+^-saturated NCS1 does not significantly alter its overall secondary structure, but still affects its EF-hands.

**FIGURE 4:**
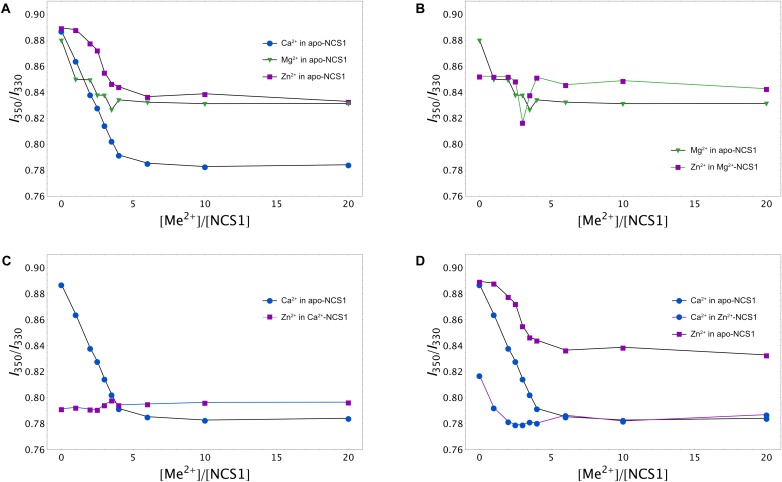
Calcium, magnesium and zinc dependences of conformational properties of NCS-1. Tryptophan fluorescence intensity at 350/330 nm (*I*_350_/*I*_330_) for apo **(A)**, Mg^2+^-saturated **(B)**, Ca^2+^-saturated **(C)**, or Zn^2+^-saturated **(D)** NCS-1 (25 μM) in the presence of increasing concentrations of the other cations. Standard deviation of *I*_350_/*I*_330_ values did not exceed 0.02.

To gain further insight into conformational differences between single and double ion-bound forms of NCS-1, we measured concentration dependencies of the ratio of its fluorescence intensities at 350 nm and 330 nm (*I*_350_/*I*_330_) for apo-NCS-1 or NCS-1, saturated by Ca^2+^, Mg^2+^ or Zn^2+^. In the presence of increasing concentrations of Mg^2+^, Ca^2+^ or Zn^2+^, a gradual reduction in the *I*_350_/*I*_330_ ratio of NCS-1 was observed, confirming interaction of the protein with these cations (Figure 4A). The decrease in the ratio was most pronounced for Ca^2+^ ions, suggesting that Ca^2+^-loaded NCS-1 is structurally different from its Mg^2+^-bound or Zn^2+^-bound states (Figure 4A). In the case of Mg^2+^-saturated NCS-1, low concentrations of Zn^2+^ decreased the *I*_350_/*I*_330_ ratio, while, at a more than threefold molar excess of zinc, the ratio increased toward the level of the Zn^2+^-bound form (Figure 4B), probably reflecting the replacement of magnesium by zinc (see previous section). In contrast, the binding of Zn^2+^ to Ca^2+^-NCS-1 produced a highly moderate increasing effect on the ratio (Figure 4C). Monitoring of the *I*_350_/*I*_330_ ratio for Zn^2+^-saturated NCS-1 in the presence of increasing calcium levels revealed signs of the Ca^2+^-bound-like conformation of the protein at lower calcium concentrations than in the case of Ca^2+^ binding to apo-NCS-1 (Figure 4D), which agreed with the increased Ca^2+^ affinity of Zn^2+^-saturated protein (see Table 1). It should be noted that differences in conformational changes induced by zinc binding to apo, Mg^2+^-saturated and Ca^2+^-saturated NCS-1 were the most striking at low zinc levels, when they were likely correlated with the stoichiometry of the metals’ binding. Thus, the *I*_350_/*I*_330_ ratio for apo-NCS-1 decreased even in the case of a onefold excess of zinc (one Zn^2+^ bound), while the same value for Ca^2+^-NCS-1 exhibited a moderate increase only when Zn^2+^ concentration exceeded the protein concentration by three times (two Zn^2+^ bound). In contrast, the fluorescence of Mg^2+^-NCS-1 remained unchanged until reaching a 2.5-fold excess of zinc, whereas the further elevation of Zn^2+^ concentration resulted in a sequential decrease (presumably one Zn^2+^ and two Mg^2+^ bound to the protein) and an increase (presumably two Zn^2+^ and one Mg^2+^ bound to the protein) in the ratio.

Summing up, the spectral measurements reveal certain structural differences between apo, Mg^2+^-bound, Ca^2+^-bound, Zn^2+^-bound, Zn^2+^(Mg^2+^)-bound, Zn^2+^(Ca^2+^)-bound and Ca^2+^(Zn^2+^)-bound conformers of NCS-1.

### Thermal Stability of NCS-1 in the Presence of Zinc

**FIGURE 5:**
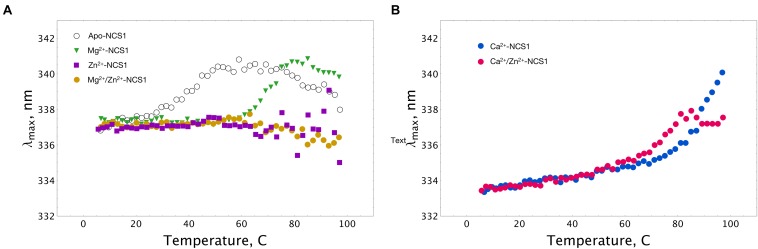
Thermal stability of NCS-1 (14 μM) under saturating levels of calcium, magnesium and zinc, monitored by tryptophan fluorescence. **(A)** Denaturation profiles of Ca^2+^-free NCS-1 in the presence of 1 mM EDTA, 1 mM Mg^2+^, 100 μM Zn^2+,^ or 1 mM Mg^2+^ and 100 μM Zn^2+^. **(B)** Denaturation profiles of Ca^2+^-bound NCS-1 (100 μM Ca^2+^) in the absence, and in the presence of 100 μM Zn^2+^. Standard deviation of λ_max_ values did not exceed 0.3 nm.

Thermal unfolding of NCS-1 is accompanied by a red shift in its tryptophan fluorescence spectrum, implying that λ_max_ can be used for monitoring thermal denaturation of the protein (Baksheeva et al., 2015). We compared the thermal unfolding profiles of NCS-1 (15 μM) in the presence of 1 mM EDTA, 1 mM Mg^2+^, 100 μM Ca^2+^, 100 μM Zn^2+^ or their combinations. Inspection of the experimental curves revealed that apo-NCS-1 was relatively unstable with a mid-transition temperature (*T*_m_) of 40°C, whereas, in the presence of magnesium, *T*_m_ increased up to 70°C (Figure 5A). In the presence of Zn^2+^, or Mg^2+^ and Zn^2+^, NCS-1 exhibited similar temperature profiles without a clear transition over the experimental temperature range (Figure 5A). In both cases, the dispersion of λ_max_ values observed at temperatures above 60^o^C indicated protein aggregation. Ca^2+^-saturated NCS-1 demonstrated blue-shifted emission spectra and a *T*_m_ value exceeding 80°C (Figure 5B). Meanwhile, zinc binding to calcium-loaded NCS-1 shifted the thermal transition of the protein toward lower temperatures, thereby reflecting structural differences between Ca^2+^-bound and Zn^2+^(Ca^2+^)-bound NCS1.

**FIGURE 6:**
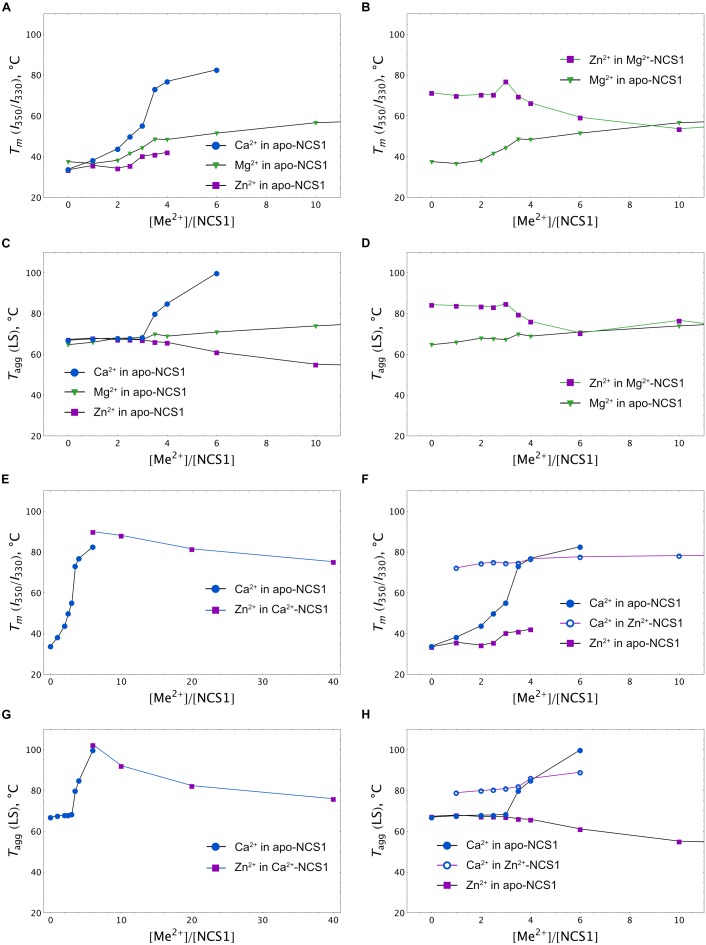
Calcium, magnesium and zinc dependences of thermal stability of NCS-1. Mid-transition temperatures of NCS-1 (25 μM) denaturation determined form tryptophan fluorescence at 350/330 nm (*I*_350_/*I*_330_) for apo **(A)**, Mg^2+^-saturated **(B)**, Ca^2+^-saturated **(E)**, or Zn^2+^-saturated **(F)** protein in the presence of increasing concentrations of the alternative cations. Mid-transition temperatures of NCS-1 (25 μM) aggregation determined form light scattering at 350 nm for apo **(C)**, Mg^2+^-saturated **(D)**, Ca^2+^-saturated **(G)**, or Zn^2+^-saturated **(H)** protein in the presence of increasing concentrations of the other cations.

More information was obtained upon monitoring NCS-1 (25 μM) denaturation by registering temperature dependences of the *I*_350_/*I*_330_ ratio at different excesses of the cations. A nanoDSF instrument was used since it allows us to monitor, in parallel, the aggregation of the protein by measuring the LS of the sample at 350 nm upon heating. The binding of any of the three cations to apo-NCS-1 increased the stability of the protein, but with different efficacy and within different concentration ranges (Figure 6A). Indeed, in the presence of the fourfold excess of calcium (100 μM), the *T*_m_ of the protein increased to > 80°C, while, in the case of the same Mg^2+^ and Zn^2+^ concentrations, the increase was moderate (48 and 42°C, respectively). Interestingly, the use of higher calcium or magnesium concentrations further improved protein stability without affecting the aggregative state, whereas zinc, at more than a fourfold excess, increased susceptibility of the protein to aggregation as indicated by LS (Figure 6C). The binding of zinc to Mg^2+^-NCS-1 had no effect on its stability until a 2.5-fold excess of Zn^2+^ was used (Figure 6B). At this point, the denaturation temperature increased to 80°C and then began to drop, apparently reflecting the formation of Zn^2+^/2Mg^2+^ NCS-1 intermediate and 2Zn^2+^/Mg^2+^ NCS-1 conformer, respectively (see above). The drop was associated with a reduction in aggregation temperature (*T*_agg_), indicating increased propensity of the protein to aggregation (Figure 6D). We were technically unable to monitor the impact of low zinc concentrations (one- to fivefold excess) on Ca^2+^-saturated NCS-1 (1 mM Ca^2+^) as *T*_m_ of the latter exceeded 90^o^C, which is beyond the detection limit of the method (Figure 6E). Yet, at higher levels, zinc produced a gradual destabilizing effect on NCS-1 and enhanced its susceptibility to aggregation (Figures 6E,G, see also Supplementary Figure S2A). Finally, the presence of Ca^2+^ inhibited aggregation of Zn^2+^-saturated NCS-1 and increased its thermal stability as soon as the protein bound the first calcium ion (onefold excess of Ca^2+^). However, at high calcium concentrations, *T*_m_ did not exceed 78^o^C, indicating that the resulting NCS-1 conformer represents a Ca^2+^(Zn^2+^)-bound form rather than a Ca^2+^-saturated form of the protein (*T*_m_ > 90^o^C) (Figures 6F,H).

Overall, at low zinc concentrations, corresponding to full saturation of Zn^2+^-binding sites in each of the NCS-1 forms, the binding of the cation slightly destabilizes Ca^2+^-loaded NCS1 and enhances the thermal stability of Ca^2+^-free protein. Meanwhile, upon elevation of Zn^2+^ levels, all NCS-1 forms become gradually destabilized and prone to aggregation. Ca^2+^-NCS-1 is the most resistant to the destabilizing effects of high zinc. Consistently, the binding of calcium to Zn^2+^-saturated NCS-1 improves its structure by forming a relatively stable Ca^2+^(Zn^2+^)-bound conformer.

### Putative Zinc-Binding Sites in Calcium-Saturated NCS-1

**FIGURE 7:**
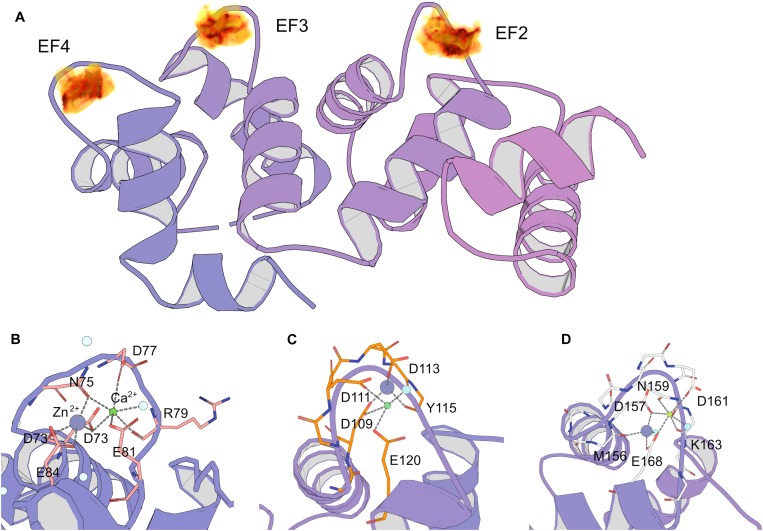
Prediction of zinc-binding sites in Ca^2+^-saturated NCS-1 using molecular modeling. **(A)** Density of putative Zn^2+^ positions in NCS-1 from geometry search visulated as volumetric data, from low (yellow) to high (black) values. **(B–D)** Positions of chelators for zinc and calcium in Ca^2+^-binding loops of EF2 **(B)**, EF3 **(C)** and EF4 **(D)** according to QM/MM molecular dynamics simulations.

The results of spectroscopic and thermal stability studies revealed the existence of a Zn^2+^(Ca^2+^)-bound form of NCS-1, which structurally differs from the well-recognized Ca^2+^-saturated conformer of the protein. Therefore, we next attempted to predict Zn^2+^-binding site locations in Ca^2+^-NCS-1 *in silico*, based on the available crystal structure of this form of the protein [PDB 5AEQ (Pandalaneni et al., 2015)]. Considering the averaged geometry of zinc coordination in all Zn^2+^-binding proteins presented in PDB, the density of Zn^2+^-binding probability in the NCS-1 structure was built in grid with a step of 0.1 Å (Figure 7A). It was found that areas with the required number of chelating groups for Zn^2+^ was located only in the loops of three functional EF-hand sites, namely EF2 (the highest score), EF3 and EF4. Interestingly, the size of these areas was around 4.5 Å, suggesting that they could simultaneously accommodate calcium and zinc ions. Furthermore, such a configuration would compensate for a negative charge (-2 in EF2, -1 in EF3 and EF4), which remained in EF hand loops upon binding of single Ca^2+^. In order to check this suggestion, we performed QM/MM simulations of molecular dynamics associated with Zn^2+^ binding in each Ca^2+^-occupied EF-hand motif. It was found that, in EF2, the number of coordinators around calcium ions in the presence of zinc decreased from seven to six, but most of the metal-chelating residues of the loop (Asp 73, Asn 75, Asp77, Arg79, Glu 81), as well as a water molecule, remained involved in the binding. In this case, the coordination of zinc was maintained by four oxygen atoms from Asp 73 (two atoms, from α-carbonyl and β-carboxyl groups), Asn 75 and Glu 84 (Figure 7B). In EF3, calcium lost three chelators coordinated by Asp109, Aps 111, Glu120 and a water molecule, whereas zinc possessed less favorable coordination due to three oxygen atoms from Tyr115, Asp 109 and Asp 111 (Figure 7C). As for EF4, both cations bound simultaneously in relatively optimal coordination. Thus, calcium was chelated by Glu168, Asp157, Asn159, Asp161, Lys163 (backbone) and a water molecule, whereas zinc was bound to Met156 (backbone), Asp157, Glu168 and a water molecule (Figure 7D). Taken together, these data indirectly support our suggestion of zinc coordination in EF2, EF3 and EF4 in apo and Mg^2+^-bound NCS-1, as well as provide a rationale for the prediction of Zn^2+^-binding sites in the second and fourth EF-hands of the Ca^2+^-saturated form of the protein.

### Functional Properties of NCS-1 in the Presence of Zinc

Previous *in vitro* and *in vivo* studies revealed that NCS-1 can regulate a number of targets including membrane-associated proteins. Consistently, an important feature of NCS-1 is its Ca^2+^-induced interaction with cellular membranes via the N-terminal myristoyl group of the protein (Baksheeva et al., 2015). Thus, we next explored whether zinc binding affects the affinity of NCS-1 to photoreceptor membranes. Among the detected forms of protein, we focused on the Zn^2+^(Mg^2+^)-bound and Zn^2+^(Ca^2+^)-bound NCS-1 conformers as they might dominate under physiological conditions. According to the data from the modified equilibrium centrifugation assay, Ca^2+^-saturated NCS-1 (25 μM) at 25^o^C in 20 mM Tris-HCl pH 8.0 buffer, bound to urea-washed photoreceptor membranes, and the binding decreased approximately twofold in the case of Ca^2+^-free/Mg^2+^-saturated NCS-1. Meanwhile, the presence of 0–100 μM Zn^2+^ did not affect the membrane association of both NCS-1 forms (Supplementary Figure S1).

**FIGURE 8:**
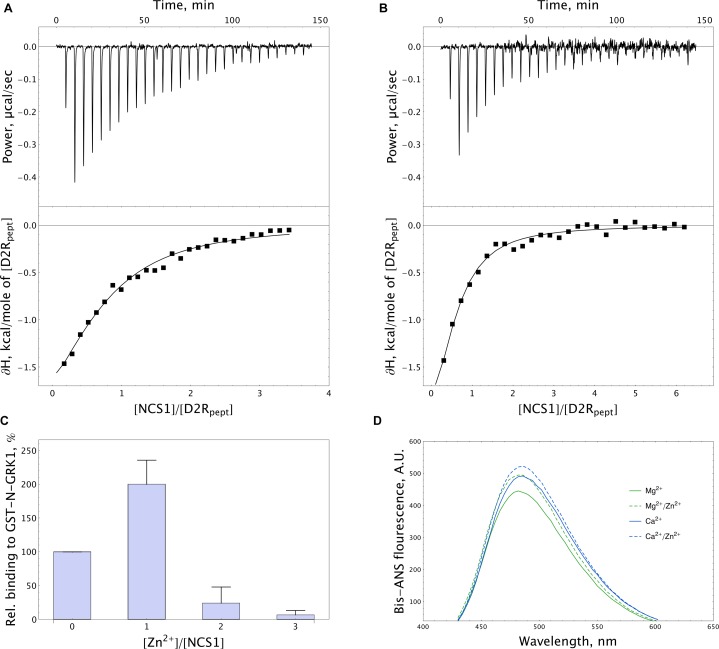
Target-binding properties of NCS-1 in the presence of zinc. **(A,B)** Typical ITC curves (upper panels) and binding isotherms (lower panels) representing titration of 50 μM D2R peptide with 5–150 μM NCS-1 in the presence of 5 mM Ca^2+^ **(A)** or 5 mM Ca^2+^ and 100 μM Zn^2+^ **(B)**. **(C)** Binding of 25 μM NCS-1 to GST-N-GRK1 at 1 mM Ca^2+^ in the presence of 0, 25, 50, or 75 μM Zn^2+^ (i.e., at [Zn^2+^]/[NCS-1] ratio of 0-3), monitored by pull-down assay. **(D)** Representative fluorescence spectra of bis-ANS (1.2 μM) and NCS-1 (5 μM) complexes formed in the presence of either 1 mM Mg^2+^ or 100 μM Ca^2+^ with or without addition of 100 μM Zn^2+^.

In order to further address the possible effects of zinc on the functional activity of NCS-1, we monitored the interaction of the protein, with D2R and GRK1 representing its well-established Ca^2+^-dependent targets (Pandalaneni et al., 2015). Firstly, the binding of NCS-1 to the complementary D2R peptide N430-R443 (50 μM) was monitored at 25^o^C in 20 mM Tris-HCl pH 8.0 buffer using ITC. Without calcium, no interaction between NCS-1 and the peptide was registered, regardless of the presence of zinc (data not shown). Meanwhile, Ca^2+^-loaded NCS-1 bound two moles of D2R peptide with a dissociation constant of 30.12 μM (Figure 8A). Remarkably, in the presence of zinc, Ca^2+^-NCS-1 interacted with D2R peptide with the same stoichiometry, but with a 3.5-fold increase in affinity (Figure 8B and Table 2). Similar observations were made upon monitoring the interaction of NCS-1 (25 μM) with N-terminal domain of GRK1 (M1-G183), fused with glutathione-S-transferase (GST-N-GRK1) at 25^o^C in 20 mM of Tris-HCl pH 8.0 buffer, by means of a pull-down assay. Thus, 1 mM Ca^2+^ GST-N-GRK1 was bound to NCS-1 and the binding was enhanced twofold in the presence of 25 μM of Zn^2+^ (Figure 8C). Interestingly, a further increase in zinc concentration resulted in the gradual destabilization of the NCS-1-GRK1 complex.

**Table 2:** Thermodynamic parameters of binding of D2R peptide to NCS-1 in 20 mM Tris-HCl buffer (pH 8.0), 150 mM NaCl, 5 mM CaCl_2_ in the presence or in the absence of 100 μM ZnCl_2_.

	Ca^2+^	Ca^2+^+Zn^2+^
N	0.676 ± 0.091	0.508 ± 0.102
K_A_, M^-1^	(3.320 ± 0.568) × 10^4^	(11.800 ± 2.550) × 10^4^
K_D_, M	30.12 × 10^-6^	8.47 × 10^-6^
ΔH, kcal M^-1^	-3.0 ± 0.5	-3.2 ± 0.8
ΔS, cal K^-1^ M^-1^	10.6	12.4
ΔG, kcal M^-1^	-6.2	-6.9


NCS-1 is known to interact with D2R and GRK1 via hydrophobic sites, which become available in response to Ca^2+^ binding (Pandalaneni et al., 2015). Therefore, we next investigated effects of zinc on the accessibility of such sites in Mg^2+^-saturated and Ca^2+^-saturated NCS-1, using fluorescent dye bis-ANS. The interaction of bis-ANS with hydrophobic cavities of a protein is accompanied by increased intensity and a shift in the λ_max_ of the fluorescence of the dye. It was found that, in the presence of zinc, bis-ANS binding to both Mg^2+^-saturated and Ca^2+^-saturated NCS-1 was moderately enhanced, suggesting increased surface hydrophobicity of these forms (Figure 8D). Thus, it is this effect that may partially account for the increased affinity of Zn^2+^(Ca^2+^)-bound NCS-1 to D2R and GRK1.

Taken together, our data demonstrate that, at low physiological concentrations, zinc cannot substitute calcium in relation to NCS-1 activation; rather, it affects the structure and stability of Ca^2+^-saturated protein, thereby improving its normal functionality.

### Abnormal Behavior of NCS-1 in the Presence of Excessive Zinc Concentrations

Although the estimated intracellular concentration of free zinc is considerably low, it is entirely possible that, under certain pathological conditions, it can abnormally increase. As such, we further analyzed behavior of different forms of NCS-1 in the presence of “pathological” amounts of zinc. According to LS data, the susceptibility of the protein to aggregation, in the presence of high zinc concentrations, decreased in the following order: apo-NCS-1 > Mg^2+^-bound NCS-1 > Ca^2+^-bound NCS-1 (Supplementary Figure S2A). For apo and Mg^2+^-bound NCS-1, the decrease in the temperature of aggregation started when Zn^2+^ concentration exceeded the concentration required for full saturation of the protein by 25 μM. In contrast, Ca^2+^-bound NCS-1 can sustain up to 100 μM free Zn^2+^. At physiological temperatures, the signs of aggregation of apo and Mg^2+^-bound NCS-1 were observed at 300–500 μM free Zn^2+^ (data not shown). Meanwhile, in the presence of calcium, no NCS-1 aggregation was detected under these conditions. Since aggregation includes multimeric associations, which can produce insoluble precipitates of the protein, we also monitored the Zn^2+^-induced precipitation of Mg^2+^-loaded and Ca^2+^-loaded NCS-1 (25 μM) at 25°C (Supplementary Figure S2B). The formation of NCS-1 precipitates was initiated at 200 and 325 μM of free zinc for Mg^2+^-loaded and Ca^2+^-loaded NCS-1 forms, respectively. To visualize the shape and arrangement of the insoluble NCS-1 conglomerates formed in the presence of zinc, we further examined the respective protein precipitates by means of TEM. As can be seen from TEM data (Supplementary Figures S2C,D), Zn^2+^-bound NCS-1 constitutes fibrilic twisted rope-like structures resembling the aggregates of another neuronal protein, TDP-43, found in the presence of zinc ions (Garnier et al., 2017).

We concluded that, at high concentrations, zinc might bind to NCS-1 non-specifically, thereby deteriorating the structure of the protein and promoting its aggregation and precipitation, which are most prominent in the absence of calcium.

## Discussion

Previous *in vitro* studies reported the existence of three major forms of NCS-1 in terms of metal binding, namely, apo, Mg^2+^-bound and Ca^2+^-bound. Meanwhile, the data concerning the stoichiometry and affinity of calcium binding to the protein are contradictory. Thus, according to flow dialysis, non-myristoylated NCS-1 (nNCS-1) cooperatively binds two calcium ions with nanomolar and micromolar affinities (Cox et al., 1994). Subsequent ITC experiments also suggested the binding of two Ca^2+^, but in a non-cooperative manner and with a dissociation constant of 1.8 μM for both sites (Jeromin et al., 2004). Meanwhile, refinement of the data using NMR studies revealed that nNCS-1 actually coordinates three calcium ions in EF2-EF4 (Chandra et al., 2011; Heidarsson et al., 2012). Myristoylated NCS-1 (mNCS-1) was reported to bind three calcium ions. However, two sets of ITC studies conducted by the same authors report different modes of calcium binding, which likely depend on the preparation of the protein samples and the model applied for fitting of the ITC data (Jeromin et al., 2004; Aravind et al., 2008). Thus, in the first study, the use of a “three sequential binding sites” model revealed binding constants of a micromolar, nanomolar and submicromolar order (Jeromin et al., 2004), whereas, in the second study, the “two sets of sites” model was applied, which allowed for identifying two similar sites with submicromolar affinity and one site with nanomolar affinity (Aravind et al., 2008). The ITC data on Ca^2+^ binding to mNCS-1, obtained in our current study, are generally in agreement with the data reported by Jeromin et al. (2004) including the revealed positive enthalpy of Ca^2+^ binding to the low-affinity site. Thus, we confirmed different calcium affinities of three EF-hands of mNCS-1 and the sequential mode of their filling (Figure 2 and Table 1). Such a mechanism agrees with previous NMR studies, according to which Ca^2+^-binding sites become occupied in the following order EF2→EF3→EF4 (Chandra et al., 2011). Considering the evaluations of the Ca^2+^ affinity of individual EF hands reported by Chandra et al., we can attribute calcium-binding constants K_A_^1^, K_A_^2^ and K_A_^3^, as calculated in the current work (Table 1), to EF3, EF2 and EF4, respectively.

In early magnesium binding experiments, nNCS-1 exhibited the non-cooperative coordination of two Mg^2+^ with a dissociation constant of 12 μM (Cox et al., 1994). Similar findings were reported for mNCS-1, based on ITC, NMR and mutagenesis studies (Aravind et al., 2008). According to our ITC data, the amount of Mg^2+^ bound to mNCS-1 tends toward three (Table 1). We speculate that such stoichiometry is a specific feature of myristoylated protein, where Mg^2+^ binds to EF2-EF4. It should be noted that the actual amount of protein-associated magnesium, which binds with low affinity, might be highly sensitive to the quality of the protein sample (i.e., the content of the nNCS-1 admixture or residual calcium) and may therefore be differently evaluated. Yet, all three studies including ours agree that Mg^2+^ antagonizes Ca^2+^ binding by reducing the affinity of the respective sites of the protein. These data confirm competition between the ions for the same binding sites with a preference for calcium (Cox et al., 1994; Aravind et al., 2008).

Our brand-new finding is that myristoylated NCS-1 is capable of coordinating up to three zinc ions. The mechanism of zinc binding to the protein and the exact Zn^2+^-binding sites are yet to be determined. For the moment, based on our metal competition analysis, CD studies and molecular modeling, we can hypothesize that zinc binds to functional EF-hands of the protein. Indeed, the ability of EF-hands to coordinate Zn^2+^ was previously reported for another ubiquitous Ca^2+^-binding protein calmodulin by X-ray crystallographic studies (Warren et al., 2007). Based on the analysis of all Zn^2+^-binding proteins presented in PDB, we found that, in NCS-1, the density of chelating groups required for Zn^2+^ binding is located only in the loops of EF2 (the highest score), EF3 and EF4 (Figure 7A). According to our CD measurements, the interaction of zinc with apo-NCS-1 induces a decrease in the content of β-sheets and an increase in α-helical content, exactly as in the case of the binding of Ca^2+^ or Mg^2+^ to EF-hands (Figure 3D and Supplementary Table S1). Since NCS-1 contains only two short antiparallel β-sheets, which connect Ca^2+^-binding loops of EF1-EF2 and EF3-EF4 (Heidarsson et al., 2012; Pandalaneni et al., 2015), one can suggest that zinc binds to EF-hands of the protein. This conclusion is further supported by our ITC data, indicating that Zn^2+^-saturated NCS-1 does not bind magnesium and exhibits reduced stoichiometry of Ca^2+^-binding (Figure 2 and Table 1). Interestingly, Ca^2+^ binding to one of the sites in Zn^2+^-saturated NCS-1 is one order of magnitude higher in affinity than any of the sites in apo-NCS-1 (Table 1). Given the proposed model for sequential filling of the mNCS-1 by calcium in the order EF2→EF3→EF4 (Chandra et al., 2011), we hypothesize that Zn^2+^-bound EF2 may adopt a conformation that facilitates the binding of calcium to the remaining two sites. Consistently, Zn^2+^-bound EF-hands of calmodulin resembled an intermediate state in the chain of conformational transitions induced by Ca^2+^-binding (Warren et al., 2007).

The unique mode of zinc binding to Ca^2+^-saturated NCS-1 is predicted by QM/MM simulations of the associated molecular dynamics, based on the crystal structure of the respective NCS-1 form [PDB 5AEQ (Pandalaneni et al., 2015)]. In the absence of zinc, EF3 possesses the most favourable environment for the coordination of calcium among EF-hands of the protein, which agrees with its maximal affinity for Ca^2+^ (Chandra et al., 2011). At the same time, coordination of both cations in this site seems unlikely due to the absence of the required number of chelating groups (Figure 7C). Therefore, EF3 can bind strictly to one ion with a preference for calcium and the latter can replace zinc from the site but not *vice versa*. In contrast, EF2 and EF4 can accommodate both Ca^2+^ and Zn^2+^, at least under our *in silico* conditions. In both cases, calcium loses one chelator, in turn becoming coordinated by six oxygen atoms (Figures 7B,D). Yet, such a configuration is common for proteins (Pidcock and Moore, 2001). Furthermore, such a configuration would completely compensate for the high negative charge in the EF2 (-2 in Ca^2+^-bound NCS-1). Thus, based on these observations, we suggest that Ca^2+^-saturated NCS-1 can accommodate up to two zinc ions, one in EF4 and the other one in EF2.

The proposed binding modes for Zn^2+^ and Ca^2+^ are generally in agreement with our ITC and spectroscopic data. Thus, Ca^2+^-saturated NCS-1 coordinates one or two zinc ions (Table 1), apparently in terms of EF4/EF2 yielding the Zn^2+^(Ca^2+^)-bound protein form, which does not significantly differ from the “open” Ca^2+^-bound conformer in the overall protein fold (Figure 4C) but possesses enhanced thermal stability (Figures 5B, 6E–H). In contrast, Zn^2+^-saturated NCS-1 coordinates two calcium ions (Table 1) yielding a Ca^2+^(Zn^2+^)-bound conformer. In this case, EF2 likely remains occupied by zinc, which could facilitate calcium binding to EF3 (and consequently to EF4) as suggested by the absence of a low-affinity Ca^2+^-binding site and an increased binding constant for the high-affinity site in the ITC data (Table 1). At the same time, calcium replaces zinc from EF3 and could replace or temporary co-reside with zinc in EF4. It cannot be ruled out, however, that one of the EF-hands, being occupied with zinc, might adopt a conformation that is favorable for calcium binding, thereby exhibiting increased Ca^2+^ affinity as seen in our ITC studies. In any case, the resulting Ca^2+^(Zn^2+^)-bound conformer possesses only a small difference with the Zn^2+^(Ca^2+^)-bound from of the protein in the *I*_350_/*I*_330_ ratio (Figures 4C,D), but significantly differs from it in thermal stability (> 20°C, Figures 6E,F). It should be emphasized that, despite being highly consistent with the experimental and literature data, the above mechanisms of Zn^2+^/Ca^2+^ binding are mostly speculative and require additional confirmations.

In the aggregate, our *in vitro* studies suggest the existence of Zn^2+^-bound, Zn^2+^(Mg^2+^)-bound, Zn^2+^(Ca^2+^)-bound and Ca^2+^(Zn^2+^)-bound conformers of NCS-1 in addition to previously recognized apo, Mg^2+^-bound and Ca^2+^-bound forms of the protein. It should be mentioned that structural differences between the two latter forms, as observed in this study, are generally in accord with the reported data. Thus, the binding of both Mg^2+^ and Ca^2+^ increases the α-helical content of NCS-1, whereas only Ca^2+^ binding notably increases its surface hydrophobicity, as originally described by Jeromin et al. (2004). In addition, Mg^2+^ binding induced a more pronounced increase in the intensity of intrinsic fluorescence of the protein than Ca^2+^ binding, in agreement with previous observations (Aravind et al., 2008). It has been suggested that the unique mode of Ca^2+^/Mg^2+^ binding and resulting structural alterations govern the target recognition by NCS-1. Indeed, the NCS-1, preloaded with Mg^2+^, binds D2R in response to Ca^2+^ elevation more efficiently when compared to apo-protein, indicating that magnesium can serve as a physiological co-factor with calcium in this interaction (Woll et al., 2011).

Alongside NCS-1, the Ca^2+^/Mg^2+^ interplay was shown to regulate the structure and function of the other NCS proteins belonging to all five classes of the NCS family. Interestingly, the mechanisms of this regulation are quite distinct. Thus, magnesium and calcium bind to different EF-hand motifs of these proteins and the binding differently affects their functional specificity. In recoverin, Mg^2+^ binds to functional EF2 and EF3, which reduces the Ca^2+^ affinity of the protein (at high magnesium concentration), but only slightly affects its secondary and tertiary structure, does not lead to activation of its myristoyl switch and is not required for its interaction with GRK1 (Ozawa et al., 2000; Ames et al., 2006; Marino et al., 2015). The cooperative sequential binding of calcium to the EF3 and EF2 of recoverin increases its thermal stability and α-helical content, as well as leads to exposure of its myristoyl group and hydrophobic pocket residues, thereby providing the protein with a capability to interact with membranes and GRK1 (Zozulya and Stryer, 1992; Ames et al., 1997, 2006; Permyakov et al., 2000; Zernii et al., 2015). A similar mechanism of Ca^2+^ binding (cooperative binding to EF2 and EF3), structural alterations (increase in α-helical content in the presence of Ca^2+^ but not Mg^2+^) and a Ca^2+^-myristoyl switch were recognized in the case of another NCS protein, VILIP1. However, unlike recoverin, VILIP1 only coordinates magnesium in EF3 with a relatively high affinity (*K*_D_ = 20 μM), suggesting the functional significance of this complex. Furthermore, VILIP-1 forms a stable dimer, which is not dependent on Ca^2+^ or Mg^2+^, but seems to be required for proper target recognition (Jheng et al., 2006; Li et al., 2011). In the proteins belonging to another class of the NCS family, GCAPs, Mg^2+^ and Ca^2+^ play a crucial role in tuning their activity toward target enzymes, i.e., retinal guanylate cyclases (GCs). For instance, in GCAP1, Mg^2+^ binds to EF2 with micromolar affinity (EF3 and EF4 exhibit only low affinity with the cation) and the binding stabilizes a tertiary structure of the protein, which otherwise represents a molten globule incapable of regulating GCs (Lim et al., 2009; Dell’Orco et al., 2010). Thus, the presence of magnesium in EF2 is necessary for maintaining a GC-activator state of GCAP1 (Peshenko and Dizhoor, 2004; Lim et al., 2016). Calcium binds to EF2, EF3 and EF4 of GCAP1 in a non-cooperative manner, which drastically increases the thermal stability of the protein without altering its secondary structure and triggering exposure of its myristoyl group (Lim et al., 2009; Marino et al., 2015). Instead, the binding converts GCAP1 into a GC-inhibitor state by inducing local conformational changes via the Ca^2+^-myristoyl tug mechanism (Peshenko et al., 2012; Lim et al., 2016). Finally, a rather different mechanism for Ca^2+^/Mg^2+^-dependent regulation was reported for NCS protein of the KChIP class, i.e., KChIP3, also known as the transcriptional repressor DREAM. In the absence of magnesium, this protein binds Ca^2+^ non-cooperatively in the following sequence EF3→EF4→EF2. Interestingly, the apo-form of KChIP3 coordinates Mg^2+^ with high affinity (*K*_D_ = 13 μM) in EF2 (EF3 and EF4 bind Mg^2+^ in the millimolar range), and this bound magnesium cannot be replaced by calcium, suggesting that, under cellular conditions, the protein will exist in either Mg^2+^-bound, or 2Ca^2+^/Mg^2+^-bound forms. Consistently, Mg^2+^-bound KChIP3 exists as a monomer and can specifically recognize target DNA elements, whereas Ca^2+^ binding to EF3 and/or EF4 induces dimerization of the protein and suppresses DNA binding. Similar to GCAP1, apo-KChIP3 represents a molten globule and Ca^2+^/Mg^2+^ binding enhances its stability (Osawa et al., 2005).

Overall, Ca^2+^/Mg^2+^ interplay governs the structural and functional properties of the majority of NCS proteins, although they exhibit different modes of regulation. Meanwhile, the involvement of zinc ions in this regulation so far has only been determined for recoverin. Similar to NCS-1, recoverin binds Zn^2+^, regardless of the presence of calcium, while the binding only slightly affects the secondary structure of the protein and destabilizes its Ca^2+^-saturated form. In recoverin, Zn^2+^ was proposed to be coordinated outside EF-hands since it binds to “inactivated” mutant with E→Q substitutions in the 12th position of the loop of functional EF2 and EF3 (E85Q/E121Q). However, this conclusion does not seem to be strict, as our current calculations indicate that E→Q mutation in such a position does not necessarily prevent the four-chelator coordination of Zn^2+^, which becomes bound by the other chelators in the loop. Consistently, such mutation does not prevent the six-chelator coordination of Mg^2+^ in EF-hands (Cates et al., 1999). Thus, it cannot be excluded that, similar to NCS1, Ca^2+^-loaded recoverin binds Zn^2+^ in one of the functional EF-hands. In this case, the reduced stoichiometry of zinc binding to NCS1 (2 Zn^2+^ per protein), compared to recoverin (1 Zn^2+^ per protein), can be explained by the fact that the latter contains a smaller amount of functional EF-hands: its EF4 is naturally non-functional due to substitutions of the metal coordinating residues in the first and third positions of the EF-hand loop. It should be noted that, unlike NCS-1, recoverin exhibits an increased affinity with photoreceptor membranes in the presence of zinc (Permyakov et al., 2003). Thus, although the coordination of Zn^2+^ may be a common property of NCS proteins, it produces somewhat different effects concerning their function, which are similar to those observed in the case of Ca^2+^ and Mg^2+^. Zinc binding may therefore additionally diversify specific regulation of NCS proteins.

It still remains an open question as to which of the discovered Zn^2+^-bound conformers of NCS-1 (see above) dominate under physiological conditions. In contrast to the well-recognized physiological role of calcium in cell signaling, zinc has long been considered as a solely structural component of proteins. Thus, being bound with picomolar to nanomolar affinities, presumably to sulfur- and nitrogen-containing ligands in tetrahedral coordination, zinc normally serves to maintain the structure and function of enzymes, transcription factors, receptors and signaling proteins (Maret and Li, 2009). According to our data, NCS-1 binds zinc transiently with a much lower affinity and likely to the sites in EF-hands. Assuming that, in neurons, the binding will occur against the background of a constantly high [up to 1-2 mM (Romani and Scarpa, 1992)] magnesium concentration and recurring elevations [up to 1 – 2 μM (Sabatini et al., 2002)] of calcium concentration, one might suggest physiological relevance only for Zn^2+^(Mg^2+^)-bound and Zn^2+^(Ca^2+^)-bound conformers of NCS-1 in addition to the well-known Mg^2+^-bound and Ca^2+^-bound forms. Nevertheless, even the formation of two additional forms might extend the functional repertoire of the protein.

Our results suggest that the binding of zinc to NCS-1 required micromolar concentration of the free cation. However, it has been generally accepted that, in contrast to magnesium and calcium, both extracellular and intracellular free zinc concentration is low. Indeed, cytosolic zinc levels are regulated by a complex Zn^2+^-buffering system and the so-called “muffling reactions”, involving buffer proteins of metallothionein (picomolar affinity with zinc) class, as well as transporters, such as ZnTs, ZIPs and DCTs, which shuttle Zn^2+^ outside the cell or into subcellular stores including mitochondria, Golgi apparatus and lysosomes (Cousins et al., 2006; Colvin et al., 2010) [for review, see (Colvin et al., 2000)]. As a result, although the total concentration of zinc in cells reaches 0.2 mM (Colvin et al., 2008), the levels of free zinc in the cytoplasm were estimated as picomolar to low micromolar (Krezel and Maret, 2006). In this case, what are the physiological conditions in which the binding of zinc to NCS-1 can occur? The growing evidence indicates that, under certain conditions, the intracellular zinc levels can transiently increase, while zinc can perform signaling functions by playing complementary signaling roles with calcium (Maret, 2001). This is especially valid for the nervous system, as it is characterized by the highest extracellular zinc concentration and Zn^2+^ is known to be specifically accumulated in neurons (Frederickson et al., 2005). The hallmark of neuronal Zn^2+^ is its neurotransmitter function, along with glutamate in so-called “gluzinergic” neurons of forebrain. In presynaptic terminals, the cation is accumulated in ZnT3-loaded synaptic vesicles and undergoes a Ca^2+^-induced release into a synaptic cleft, where it can modulate various ionotropic and metabotropic receptors. The resulting high zinc concentration in the cleft (raised from 0.5 to 300 μM) can be pumped back to the presynaptic cell by ZnT3, or permeate into the postsynaptic neurons through calcium channels, thereby increasing the local cytosolic level of the cation (Frederickson and Bush, 2001). Furthermore, under certain conditions, zinc can be released from intracellular sources. For instance, in the hippocampal neurons exposure to glutamate-induced Ca^2+^ influx triggers cytosolic acidification and intracellular Zn^2+^ release (Kiedrowski, 2012). The resulting zinc signals could be recognized by specialized Zn^2+^-binding sites in neuronal proteins in order to conduct biochemical stimuli (Maret, 2006; Maret and Li, 2009). The levels of zinc are also high in neuroretina, where the maximal amount of total zinc was found in inner segments and synaptic terminals of photoreceptors cells, suggesting that it may participate in photoreceptor metabolism and neurotransmission (Ugarte et al., 2012; Ugarte and Osborne, 2014). Similar to gluzinergic neurons, photoreceptors have been suggested as releasing zinc together with glutamate in synapses. Indeed, both neurotransmitters were found in synaptic vesicles of the outer plexiform layer, which also contained ZnT3 (Akagi et al., 2001; Ugarte and Osborne, 2014). Importantly, photoreceptors contain considerable amounts of free zinc (or so-called “loosely bound zinc”), while its concentration varies depending on the light conditions, which, in turn, are likely to generate zinc signals (Ugarte and Osborne, 2014). Our data suggest that such signals in CNS and retinal neurons can be detected and transmitted by NCS-1. In ITC studies, the presence of zinc enhances the binding of Ca^2+^-NCS-1 to D2R (Figures 8A,B and Table 2), a process known to suppress the desensitization of the receptor. Therefore, upon receiving a joint Ca^2+^/Zn^2+^ stimulus, NCS-1 can modulate dopamine signaling in a specific enhanced manner. In addition, Zn^2+^(Ca^2+^)-bound NCS-1 can specifically regulate the desensitization of D2R or other homologous receptors by GRKs. Indeed, the presence of low zinc levels improved the binding of Ca^2+^-NCS-1 to GRK1, whereas the subsequent elevations in zinc concentration produced the opposite effect (Figure 8C). Finally, Zn^2+^ binding can affect the well-recognized function of NCS-1 in the Ca^2+^-dependent regulation of neurotransmission and synaptic plasticity, as the most pronounced increase in intracellular zinc is expected to be in synaptic terminals, while NCS-1 is known to be specifically accumulated in this part of the neurons including photoreceptors (De Raad et al., 1995; Tsujimoto et al., 2002; Sippy et al., 2003; Negyessy and Goldman-Rakic, 2005).

On the other side, uncontrolled elevations of zinc in neurons and the consequential impairment of Zn^2+^ and Ca^2+^ ion interplay can produce pathological effects. One of the principal causes of these elevations is thought to be oxidative stress (Wood and Osborne, 2001; Sensi et al., 2003; Sheline et al., 2010b). Thus, in CNS neurons, zinc becomes released from metallothioneins in response to the oxidation or nitrosylation of their cysteine residues (Bossy-Wetzel et al., 2004). In photoreceptors, the major source for pathological free zinc is rhodopsin, which coordinates seven zinc ions per dimer and can lose them in response to light-induced oxidative stress (Sheline et al., 2010b). All these events have close ties to neurological and neuro-ophthalmological disorders. Thus, increased zinc concentration in postsynaptic neurons was shown to promote excitotoxic cell death after seizures and mechanical brain trauma, while zinc chelators were found to be neuroprotective (Suh et al., 2000). Consistently, exposure of neurons to high zinc concentrations or induction of intracellular zinc release promotes their apoptosis (Manev et al., 1997; Bossy-Wetzel et al., 2004). Elevations of retinal zinc are associated with ischemia, trophic deprivation or hypoglycemia leading to neuronal death (Yoo et al., 2004; Suh et al., 2008; Sheline et al., 2010a). The mechanisms of zinc toxicity generally involve massive Zn^2+^-induced aggregation of neuronal proteins (Cuajungco and Faget, 2003). For instance, an increase in extracellular zinc is a key factor in the aggregation of amyloid plaques in Alzheimer’s disease. Furthermore, overexpression of zinc transporter proteins was observed in patients in the early stages of this disorder (Lyubartseva and Lovell, 2012). In the current study, we have demonstrated that non-specific low-affinity binding of excessive zinc promotes the aggregation and precipitation of NCS-1, which are associated with the formation of fibrilic twisted rope-like structures of the protein (Supplementary Figure S2). These structures resemble the Zn^2+^-induced aggregates of another neuronal protein, TDP-43, the aggregation of which is associated with amyotrophic lateral sclerosis and frontotemporal lobar degeneration (Garnier et al., 2017). There are no direct indications concerning the involvement of NCS-1 in the pathogenesis of neurodegenerative diseases. Meanwhile, its transcription levels were found to be altered in Alzheimer’s disease, which is known to be associated with altered zinc homeostasis (Karim et al., 2014). Furthermore, a large body of evidence supports the neuroprotective role of NCS-1 (Nakamura et al., 2006; Yip et al., 2010), which might be suppressed upon the Zn^2+^-induced loss of the protein structure. In addition, it has been proposed that NCS-1 misfolding, together with calcium dysregulation, contributes to neurodegeneration (Heidarsson et al., 2014). These observations are in accord with the fact that the partial proteolytic degradation of NCS-1 and the loss of intracellular calcium signaling induce peripheral neuropathy associated with chemotherapy by paclitaxel (Boeckel and Ehrlich, 2018).

In summary, our study suggests that the complex interplay between magnesium, calcium and zinc ions results in the appearance of multiple conformations of NCS-1, thereby modulating its functional status. It also indicates that the extreme elevation of zinc levels peculiar to some neurodegenerative and neuro-ophthalmological disorders may cause the formation of unstable Zn^2+^-bound conformers of NCS-1 and promote its aggregation. Further studies are required for unraveling the molecular mechanism and exact sites of zinc binding to NCS-1 and firmly establishing of physiological and pathological roles of this phenomenon.

## Author Contributions

PT, AR, and FD performed ITC, DSF, and TEM studies. VB, AZ, and EZ performed functional assays and ITC studies. AN, MS, and SP performed fluorimetric, CD, contributed to equilibrium dialysis, and AAS experiments. VV and DZ performed expression and purification of the proteins and analytical gel-filtration. MB and AG performed molecular modeling and QM/MM molecular dynamics simulations. PT, SP, and EZ wrote the article.

## Conflict of Interest Statement

The authors declare that the research was conducted in the absence of any commercial or financial relationships that could be construed as a potential conflict of interest.

## Footnotes

1 http://www.gelanalyzer.com/

## Supplementary Material

Figure S1 Binding of NCS-1 to photoreceptor membranes in the presence of zinc. Weight fractions of NCS-1 (25 μM) bound to urea-washed bovine photoreceptor membranes in the presence of 0, 25, 50, 75, or 100 μM Zn^2+^ [i.e., at (Zn^2+^)/(NCS-1) ratio of 0-4] under Ca^2+^-free conditions (-Ca^2+^) or on the background of 1 mM Ca^2+^ (+Ca^2^**^+^**) according to the data from equilibrium centrifugation assay.

Figure S2 Structural properties of NCS-1 in the presence of excessive zinc concentrations. **(A)** Mid-transition temperatures of NCS-1 (25 μM) aggregation in the presence of 0.1–2000 μM Zn^2+^ on the background of either 1 mM Mg^2+^ or 1 mM Ca^2+^ determined form light scattering at 350 nm. **(B)** Weight fractions of NCS-1 (25 μM) precipitated in the presence of 0–500 μM Zn^2+^ on the background of either 1 mM Mg^2+^ or 1 mM Ca^2+^. **(C,D)** Electron microphotographs of NCS-1 aggregates, obtained in the presence of 5 mM Zn^2+^; scale: 0.5 μm **(C)**, 0.2 μm **(D)**.

Table S1 Secondary structure content (in %) of NCS-1 estimated from CD data obtained at 25°C (10 mM Hepes-KOH buffer (pH 7.6), 100 mM KCl) under metal-free conditions or in the presence of Ca^2+^ (100 μM CaCl_2_), Mg^2+^ (1 mM MgCl_2_), Zn^2+^ (100 μM ZnCl_2_) or their combinations.
