# Supplementary figures and images for: Functional Status of Neuronal Calcium Sensor-1 Is Modulated by Zinc Binding
Source: Front Mol Neurosci. 2018 Dec 14;11:459. doi: 10.3389/fnmol.2018.00459 (PMC6302015; doi:10.3389/fnmol.2018.00459)

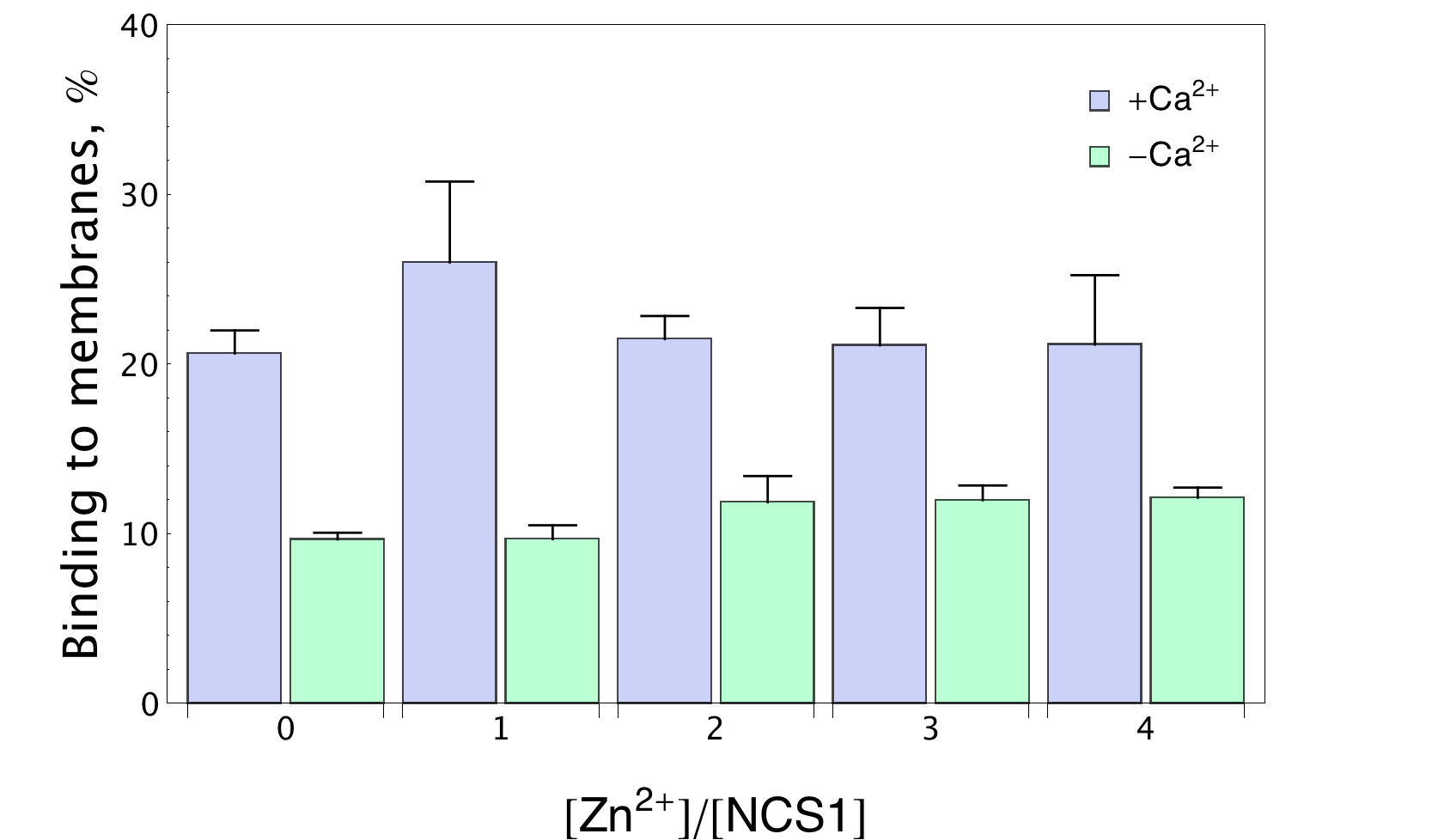

Supplement: Figure S1 — Binding of NCS-1 to photoreceptor membranes in the presence of zinc. Weight fractions of NCS-1 (25 μM) bound to urea-washed bovine photoreceptor membranes in the presence of 0, 25, 50, 75, or 100 μM Zn2+ [i.e., at (Zn2+)/(NCS-1) ratio of 0-4] under Ca2+-free conditions (-Ca2+) or on the background of 1 mM Ca2+ (+Ca2+) according to the data from equilibrium centrifugation assay. [file Image_1.TIF]

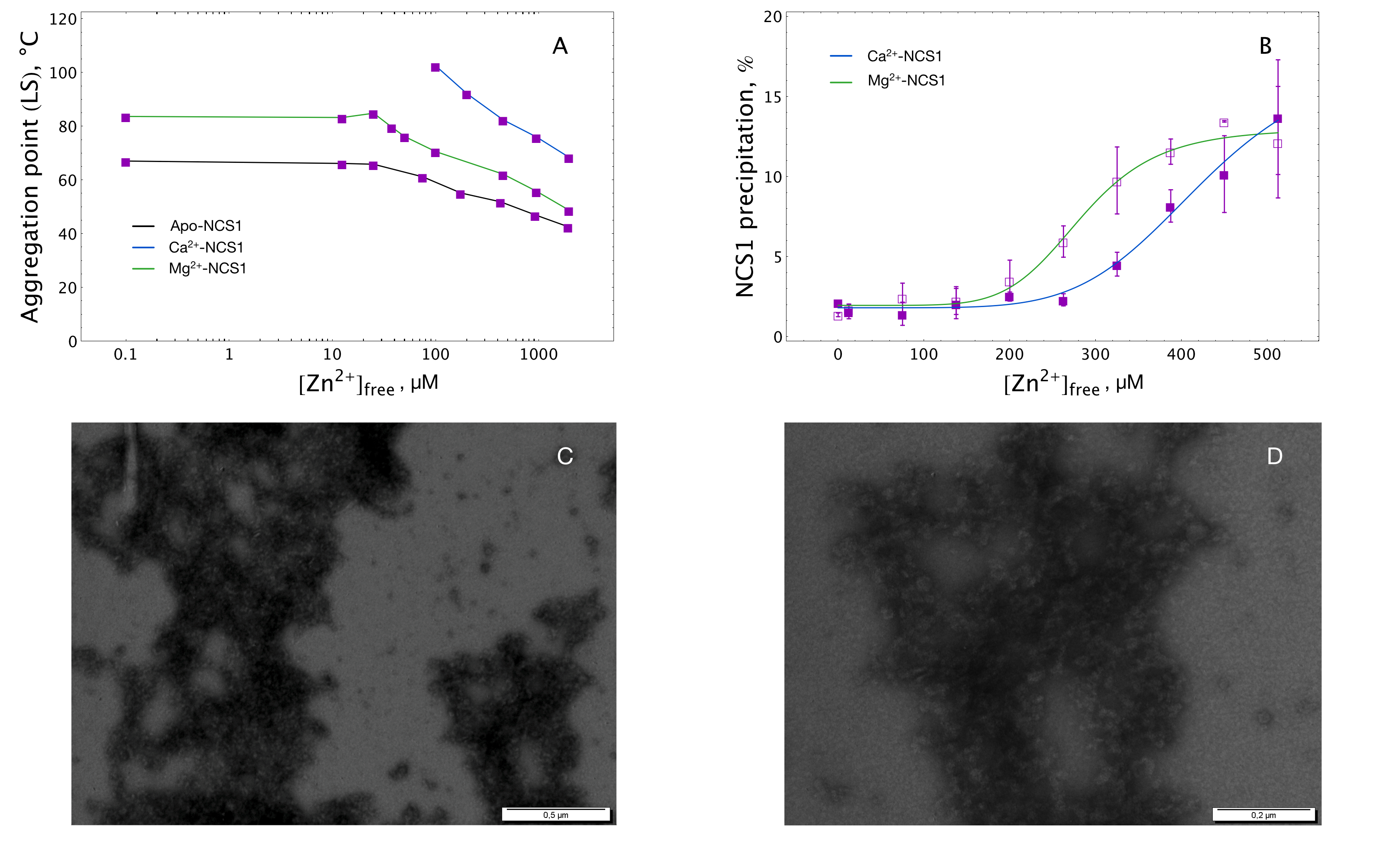

Supplement: Figure S2 — Structural properties of NCS-1 in the presence of excessive zinc concentrations. (A) Mid-transition temperatures of NCS-1 (25 μM) aggregation in the presence of 0.1–2000 μM Zn2+ on the background of either 1 mM Mg2+ or 1 mM Ca2+ determined form light scattering at 350 nm. (B) Weight fractions of NCS-1 (25 μM) precipitated in the presence of 0–500 μM Zn2+ on the background of either 1 mM Mg2+ or 1 mM Ca2+. (C,D) Electron microphotographs of NCS-1 aggregates, obtained in the presence of 5 mM Zn2+; scale: 0.5 μm (C), 0.2 μm (D). [file Image_2.TIF]
