# Supplementary material for: Functional Status of Neuronal Calcium Sensor-1 Is Modulated by Zinc Binding
Source: Front Mol Neurosci. 2018 Dec 14;11:459. doi: 10.3389/fnmol.2018.00459 (PMC6302015; doi:10.3389/fnmol.2018.00459)
Supplement: Table S1 — Secondary structure content (in %) of NCS-1 estimated from CD data obtained at 25°C (10 mM Hepes-KOH buffer (pH 7.6), 100 mM KCl) under metal-free conditions or in the presence of Ca2+ (100 μM CaCl2), Mg2+ (1 mM MgCl2), Zn2+ (100 μM ZnCl2) or their combinations. [file Table_1.DOCX]

| **Table S1**. Secondary structure content (in %) of NCS-1 estimated from CD data obtained at 25ºC (10 mM Hepes-KOH buffer (pH 7.6), 100 mM KCl) under metal-free conditions or in the presence of Ca^2+^ (100 μM CaCl_2_), Mg^2+^ (1 mM MgCl_2_), Zn^2+^ (100 μM ZnCl_2_) or their combinations. | | | | |
| --- | --- | --- | --- | --- |
| NCS-1 form | α-helices, % | β-structures, % | Turns, % | Unordered structures, % |
| Apo | 40.82±0.86 | 11.97±0.58 | 18.33±0.35 | 29.14±0.73 |
| Mg2+ | 50.68±0,86 | 6.5±0,58 | 15.9±0.35 | 26.9±0.73 |
| Ca2+ | 52.37±0.86 | 5.78±0.58 | 16.5±0.35 | 26.4±0.73 |
| Zn2+ | 48.98±0,86 | 6.35±0,58 | 18.1±0.35 | 27.0±0.73 |
| Zn2+(Mg2+) | 48.87±0,86 | 7.73±0,58 | 20.2±0.35 | 23.9±0.73 |
| Zn2+(Ca2+) | 52.43±0,86 | 8.43±0,58 | 15.9±0.35 | 24.0±0.73 |
